# An Effective Integrated Machine Learning Framework for Identifying Severity of Tomato Yellow Leaf Curl Virus and Their Experimental Validation

**DOI:** 10.34133/research.0016

**Published:** 2023-01-10

**Authors:** Nattanong Bupi, Vinoth Kumar Sangaraju, Le Thi Phan, Aamir Lal, Thuy Thi Bich Vo, Phuong Thi Ho, Muhammad Amir Qureshi, Marjia Tabassum, Sukchan Lee, Balachandran Manavalan

**Affiliations:** ^1^ Department of Integrative Biotechnology, College of Biotechnology and Bioengineering, Sungkyunkwan University, Suwon 16419, Gyeonggi-do, Republic of Korea.; ^2^ Computational Biology and Bioinformatics Laboratory, Department of Integrative Biotechnology, College of Biotechnology and Bioengineering, Sungkyunkwan University, Suwon 16419, Gyeonggi-do, Republic of Korea.

## Abstract

Tomato yellow leaf curl virus (TYLCV) dispersed across different countries, specifically to subtropical regions, associated with more severe symptoms. Since TYLCV was first isolated in 1931, it has been a menace to tomato industrial production worldwide over the past century. Three groups were newly isolated from TYLCV-resistant tomatoes in 2022; however, their functions are unknown. The development of machine learning (ML)-based models using characterized sequences and evaluating blind predictions is one of the major challenges in interdisciplinary research. The purpose of this study was to develop an integrated computational framework for the accurate identification of symptoms (mild or severe) based on TYLCV sequences (isolated in Korea). For the development of the framework, we first extracted 11 different feature encodings and hybrid features from the training data and then explored 8 different classifiers and developed their respective prediction models by using randomized 10-fold cross-validation. Subsequently, we carried out a systematic evaluation of these 96 developed models and selected the top 90 models, whose predicted class labels were combined and considered as reduced features. On the basis of these features, a multilayer perceptron was applied and developed the final prediction model (IML-TYLCVs). We conducted blind prediction on 3 groups using IML-TYLCVs, and the results indicated that 2 groups were severe and 1 group was mild. Furthermore, we confirmed the prediction with virus-challenging experiments of tomato plant phenotypes using infectious clones from 3 groups. Plant virologists and plant breeding professionals can access the user-friendly online IML-TYLCVs web server at https://balalab-skku.org/IML-TYLCVs, which can guide them in developing new protection strategies for newly emerging viruses.

## Introduction

Tomato yellow leaf curl virus (TYLCV) is one of the most notorious plants viral pathogens because it causes severe damage to tomato production globally [1,2]. TYLCV is a plant virus in the family *Geminiviridae*, belonging to the Old World *Begomovirus* genus, consisting of a single-stranded circular DNA monopartite genome (DNA-A) of approximately 2.6 to 2.8 kb encapsulated in a twinned icosahedral shape. TYLCV DNA-A currently contains 8 open reading frames (ORFs), 3 ORFs on the viral sense strand (V1, V2, and V3), and 5 ORFs on the complementary sense strand (C1, C2, C3, C4, and C5) [3–7]. DNA-A contains 180 to 200 bp of sequence within the intergenic region, including the conserved nanonucleotide sequence (TAATATTAC) stem loop, which is the viral origin of replication [8]. TYLCV is phloem-limited virus in its hosts and is transmitted by the whitefly (*Bemisia tabaci*) in a persistent and circulated manner [9]. The typical phenotypes of TYLCV-infected tomato plants’ symptoms are stunting, severe leaf curling, and yellowing. TYLCV may also be transmitted via seeds, which can cause widespread occurrence and tremendous rates of spread to new regions, countries, and continents [10]. In 1931, TYLCV was reported in the Middle East. It has since spread throughout the tropical and subtropical regions [5]. After the first isolation of TYLCV in Korea in 2008, the virus has consistently spread across the country [11,12]. There has been a TYLCV outbreak in tomato crops every year for more than 20 years. Phylogenetic analysis of TYLCV isolates from Korea revealed 2 groups; the “Masan (TYLCV-KG1)” group was most similar to the TYLCV Israel strain (GenBank: X76319) as a severe strain, and the “Jeju/Jeonju (TYLCV-KG2)” group was comparable to the Japanese group (GenBank: AB192966) as a mild strain [13].

**Fig. 1.**
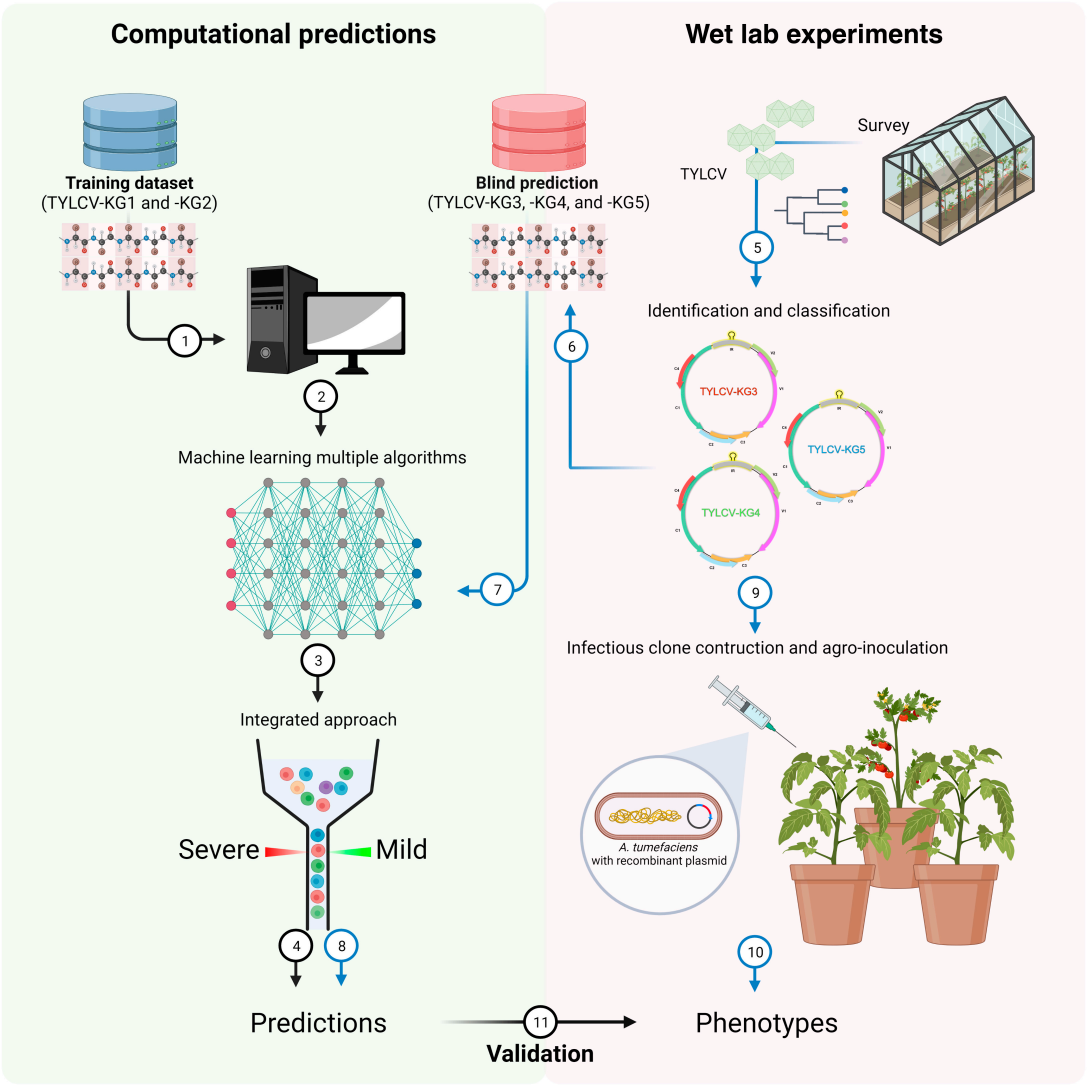
The convergence of computational predictions and wet lab experiments to validate the symptom severity of TYLCV was summarized in the following paragraphs. 1: Collection of the training dataset. 2: Generation of the training dataset using machine learning. 3: Training dataset-integrated approach. 4: Prediction results of the training dataset. 5: Survey of the TYLCV sample. 6: Identification and sequencing analysis of TYLCV. 7: Using the novel TYLCV isolates as the blind predictions for machine learning. 8: Prediction results of the novel TYLCV isolates. 9: Construction infectious clones and agro-inoculation of the novel TYLCV isolates. 10: Phenotype observation of inoculated plants. 11: Validation of the predicted TYLCV symptom severity by using plant phenotypes.

Most tomato farmers in Korea cultivate TYLCV-resistant cultivars containing different *Ty* loci because of the economic importance of TYLCVs. Several TYLCV resistance gene sources have been identified, including *Ty*-1, *Ty*-2, *Ty*-3, *Ty*-4, and *Ty*-5 [14–18]. Over the past decade, this strategy has been effective in protecting tomatoes from TYLCV infection. However, recently, TYLCV has reemerged in *Ty*-resistant cultivars of tomato. The genetic diversity of TYLCV populations may have contributed to the breakdown of resistance, which has led to the reemergence of new viruses and diseases [19]. There are various mechanisms that can result in variations in the virus population, such as mutations, inversions of nucleic acid base sequences, recombination, and mixed infections [20]. In the case of cotton leaf curl Multan virus, researchers demonstrated the comparative analysis of genetic variability and evolutionary patterns using bioinformatics-based populations, but this was not sufficient [21]. This molecular virology research combined with machine learning (ML)-based informatics may predict newly emerging viruses and viral evolution in advance [22]. According to Lalmuanawma et al. [23], the recent COVID-19 pandemic has evidenced that ML and artificial intelligence applications have helped medical experts and policymakers cope with the situation. Inspired by these studies, we conducted an interdisciplinary approach to predict the functions of novel TYLCV groups (TYLCV-KG3, TYLCV-KG4, and TYLCV-KG5) and validated our predictions through experimental tests (Fig. 1).

**Fig. 2.**
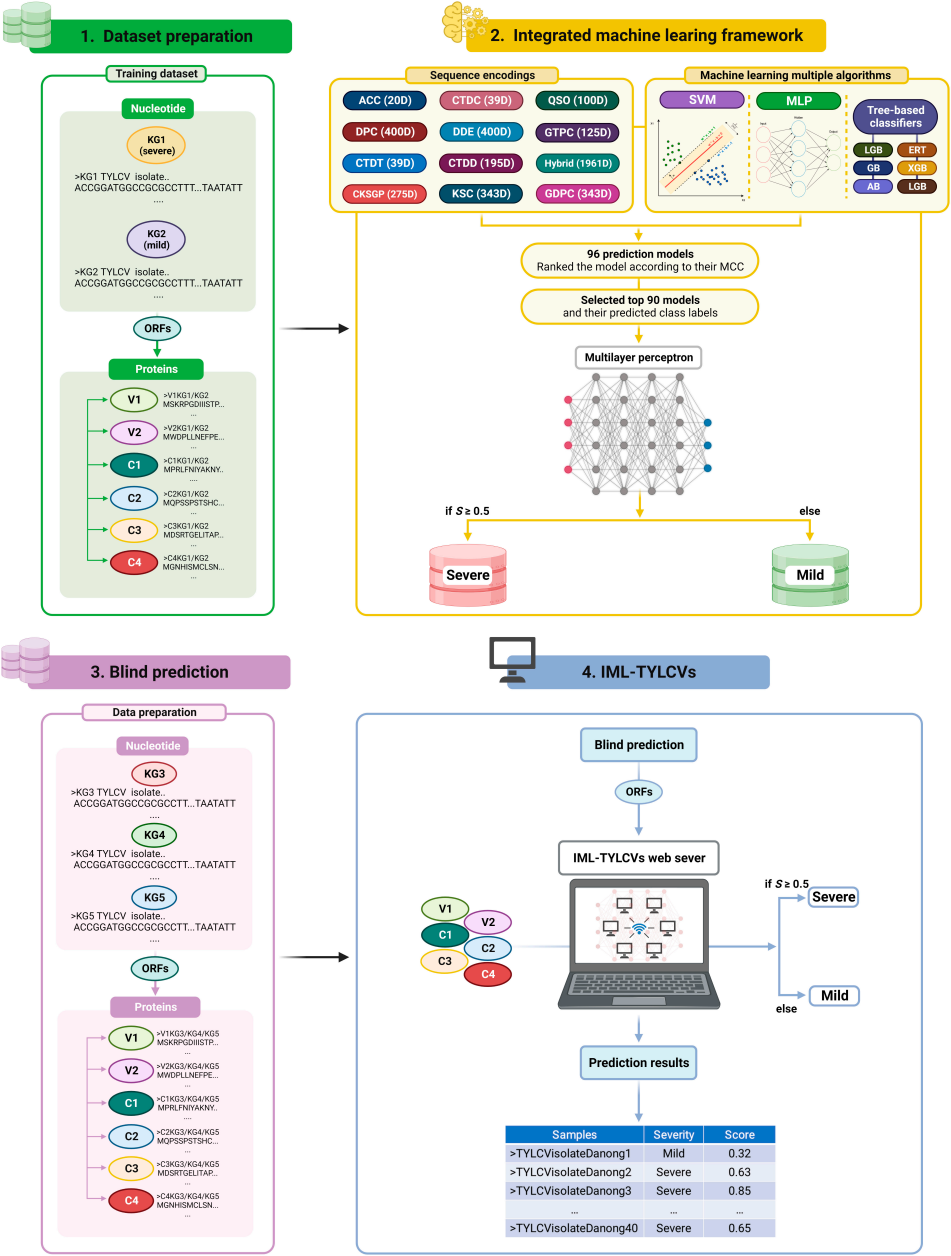
An overview of our IML-TYLCVs for predicting mild and severe strains. This figure illustrates the multiple stages involved in the construction of an integrated machine learning framework.

In this study, we developed a novel integrated ML framework for identifying the symptom severity of TYLCV from the sequence information (Fig. 2). It involves the following steps: (a) we collected TYLCV-KG1 and TYLCV-KG2 nucleotide sequences and converted them into protein sequences using the ORF finder tool. (b) We explored 12 different feature descriptors, including 11 conventional descriptors and a linear combination of all 11 descriptors (hybrid features), as well as 8 ML classifiers, including 6 tree-based classifiers, support vector machines, and multilayer perceptrons (MLP). (c) Using randomized 10-fold cross-validation, we constructed 96 prediction models and selected the top 90, whose predicted class labels were taken into consideration as reduced features through systematic analysis. These reduced features were then used to develop the final prediction model (IML-TYLCVs) using MLP (Fig. 2). Simultaneous analysis of novel TYLCV sequences extracted from *Ty*-resistant tomato cultivars showing typical TYLCV disease symptoms in Korea led to the identification of 3 novel TYLCV groups. Using the IML-TYLCVs program, we made blind predictions of symptom severity on novel TYLCVs, including TYLCV-KG3, TYLCV-KG4, and TYLCV-KG5. The results showed that TYLCV-KG3 and TYLCV-KG4 were severe strains, while TYLCV-KG5 was mild. To verify the computational prediction, infectious clones of the 3 isolates were constructed for virus-challenging experiments. The prediction was confirmed by analyzing the phenotypes of the plants, the severity of the symptoms, and the interaction between gene expressions.

## Results

### Construction of baseline models

In order to understand the similarities and differences between mild and severe strains, we conducted a compositional analysis based on the training dataset (Table S1). Figure S1A demonstrates that mild and severe strains have slightly different amino acid compositions (AAC) (*P* > 0.05). However, the dipeptide composition (DPC) compositional analysis revealed that 18 dipeptides (Fig. S1B) were significantly different between the mild and severe strains (*P* < 0.05), indicating that such differences may contribute to their differing characteristics.

**Fig. 3.**
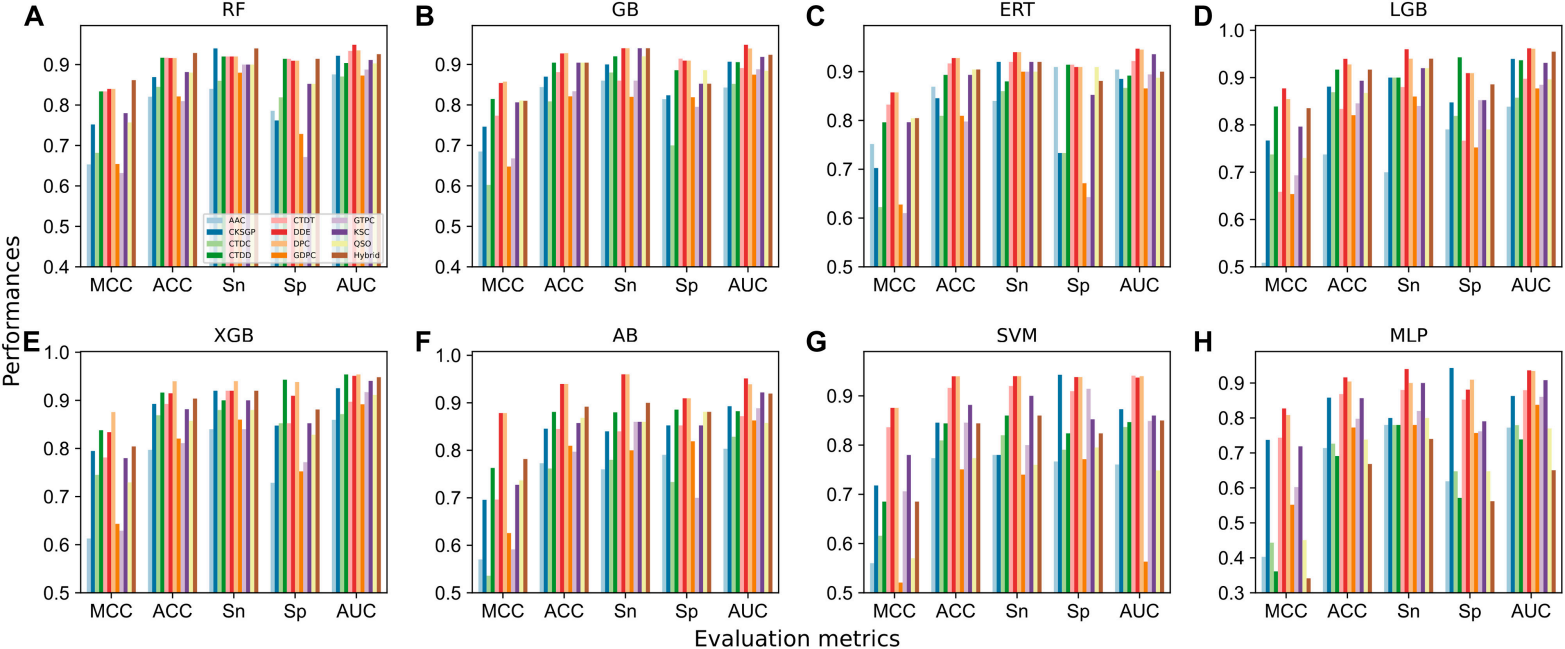
Performance comparison of 12 different encodings with respect to different classifiers. (A) Random forest (RF), (B) Gradient boosting (GB), (C) Extremely randomized tree (ERT), (D) Light gradient boosting (LGB), (E) Extreme gradient boosting (XGB), (F) Adaboost (AB), (G) Support vector machine (SVM), and (H) Multilayer perceptron (MLP). MCC, Mathew’s correlation coefficient; ACC, accuracy; Sn, sensitivity; Sp, specificity; AUC, area under the curve.

To develop the baseline models, we employed 12 different feature descriptors, including AAC, dipeptide deviation from the expected mean (DDE), quasi-sequence order (QSO), DPC, grouped DPC (GDPC), grouped tripeptide composition (GTPC), 3 different aspects of composition transition and distributions (CTDT, CTDC, and CTDD), the composition of *k*-spaced amino acid group pairs (CKSGP), *k*-spaced conjoint triad (KSC), and hybrid features (linear combination of 11 feature descriptors), and eight different ML classifiers, including random forest (RF), gradient boosting (GB), extremely randomized tree (ERT), light gradient boosting (LGB), extreme gradient boosting (XGB), Adaboost (AB), support vector machine (SVM), and MLP. Each classifier was trained 50 times using a randomized 10-fold cross-validation procedure to determine the optimal parameters. Fig. 3 illustrates the performance of the final 96 baseline models according to their optimal parameters.

The results indicate that DPC and DDE encodings achieved similar performance and ranked among the top 2 regardless of the classifiers used. However, the performance of the remaining encodings differed between classifiers. A hybrid feature achieved the best performance while using RF (accuracy (ACC) of 0.929); however, when using MLP, the performance substantially decreased (0.688). Similarly, CTDT encoding performed better when SVM and ANN were employed (0.916 and 0.888, respectively) but deteriorated when LGB was used (0.834). It appears that the necessity of experimenting with different classifiers on each encoding set is vital to understanding ML behavior and possibly selecting the best algorithm. Overall, RF, ANN, LGB, AB, ERT, GB, SVM, and XGB achieved the best area under the curve (AUC) values of 0.926, 0.936, 0.962, 0.951, 0.947, 0.939, 0.940, and 0.954, respectively. In general, one of these models has been selected and considered as the final model. However, we utilized all baseline models that had an ACC of greater than 70% in order to develop a more reliable and robust model.

### Development of IML-TYLCVs

**Fig. 4.**
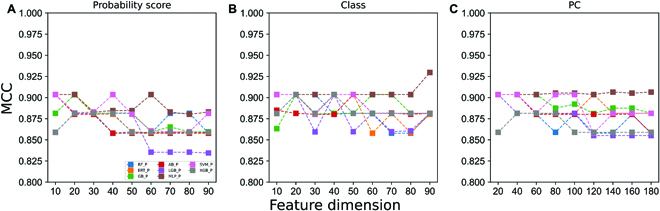
Performance of different classifiers based on (A) probability scores, (B) class information, and (C) their combination (PC). The *X* axis represents the feature dimension, and the *Y* axis represents the performance as expressed by MCC.

The baseline models were ranked according to the Mathew’s correlation coefficient (MCC), and the top 10 to 90 models were selected with a 10-model interval. Each baseline model is capable of predicting class scores and probability scores for severe and mild cases. Therefore, we considered class label information, probability score (severe), and their combination (probability and class (PC)) separately, thus obtaining 3 different groups. In each group, there are 9 different feature dimensions, each of which is input into 8 different classifiers, and the corresponding prediction models are developed using 50 randomized 10-fold cross-validation. A comparison of the performance of different classifiers using probabilistic score features, class labels, and PC information is presented in Figs. S2 to S4, respectively. In order to provide an overview of the comparison between these models, we compared them in terms of their MCC, as shown in Fig. 4. For all 3 groups, the majority of the classifiers reached their peak performance within 40D features and then began to deteriorate as the features were added. In the case of class labels, MLP performance remains stable and reached its peak with an MCC of 0.930, and the corresponding feature dimension is 90. It is noteworthy that the MLP-based model outperforms 215 other models. Therefore, we selected this model as the final model and named it as IML-TYLCVs.

Furthermore, we used the IML-TYLCVs server to perform blind predictions on the TYLCV-KG3, -KG4, and -KG5 sequences. The analysis of individual sequence predictions will not be useful for drawing conclusions about each group. To determine whether a strain is severe or mild, we computed an average predicted probability score (severe) from all the sequences related to each group and utilized the standard cutoff of 0.5. The predicted scores for IML-TYLCVs for TYLCV-KG3 and -KG4 are 0.524 (severe) and 0.695 (severe) respectively, whereas for TYLCV-KG5 is 0.195 (mild).

### Comparison of IML-TYLCVs with the top 5 baseline models on training and blind predictions

**Fig. 5.**
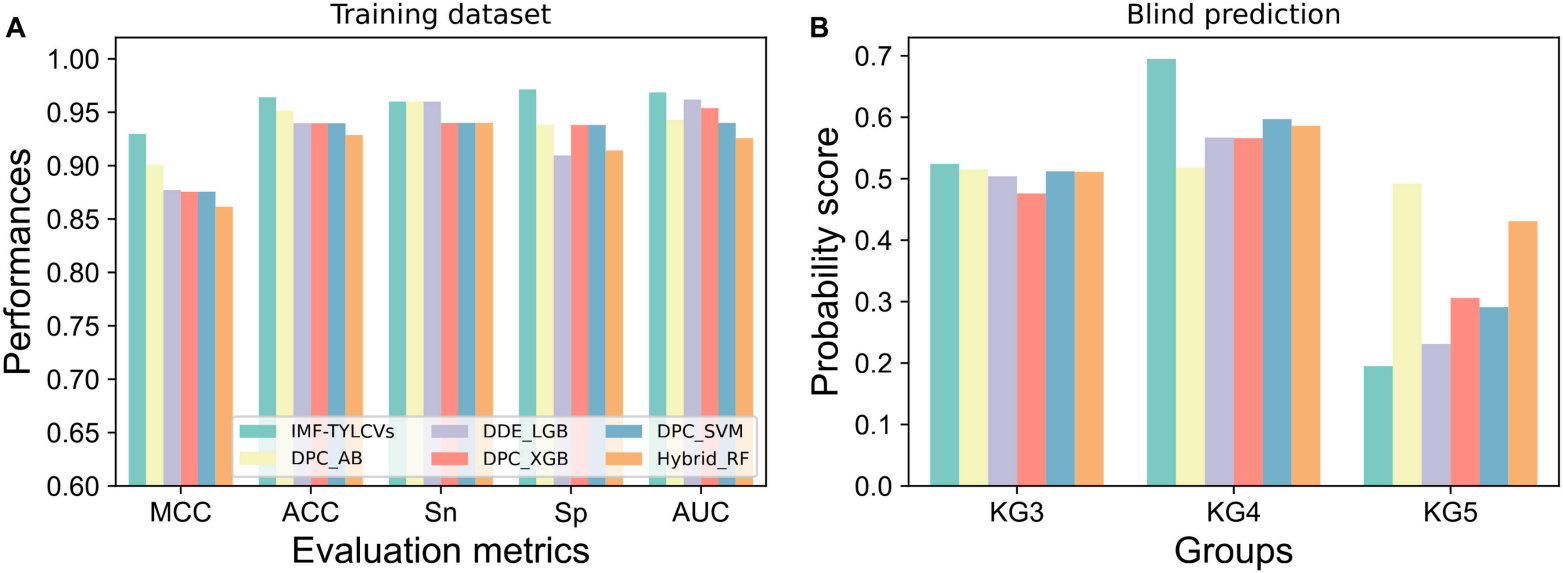
Performance comparison between IML-TYLCVs and the top 5 baseline models on training dataset (A) and blind predictions (B).

In the training, IML-TYLCVs achieve MCC, ACC, sensitivity (Sn), specificity (Sp), and AUC values of 0.930, 0.964, 0.960, 0.9714, and 0.969, respectively (Fig. 5A). In particular, the MCC of IML-TYLCVs increased by 2.9% to 6.22%, ACC by 1.25% to 3.53 %, and AUC by 0.71% to 4.33% compared to the top 5 baseline models, demonstrating that an integrated approach based on a systematic analysis has improved prediction accuracy. In the case of blind predictions, the prediction outcomes are the same across all models; however, the predicted probability scores differ significantly across these 6 models. Compared to baseline models, IML-TYLCV generated slightly higher probability scores for TYLCV-KG3, significantly higher scores for TYLCV-KG4, and significantly lower scores for TYLCV-KG5 (Fig. 5B), suggesting that integrated approaches can generate higher confidence when making decisions (severe/mild).

### Model interpretation

**Fig. 6.**
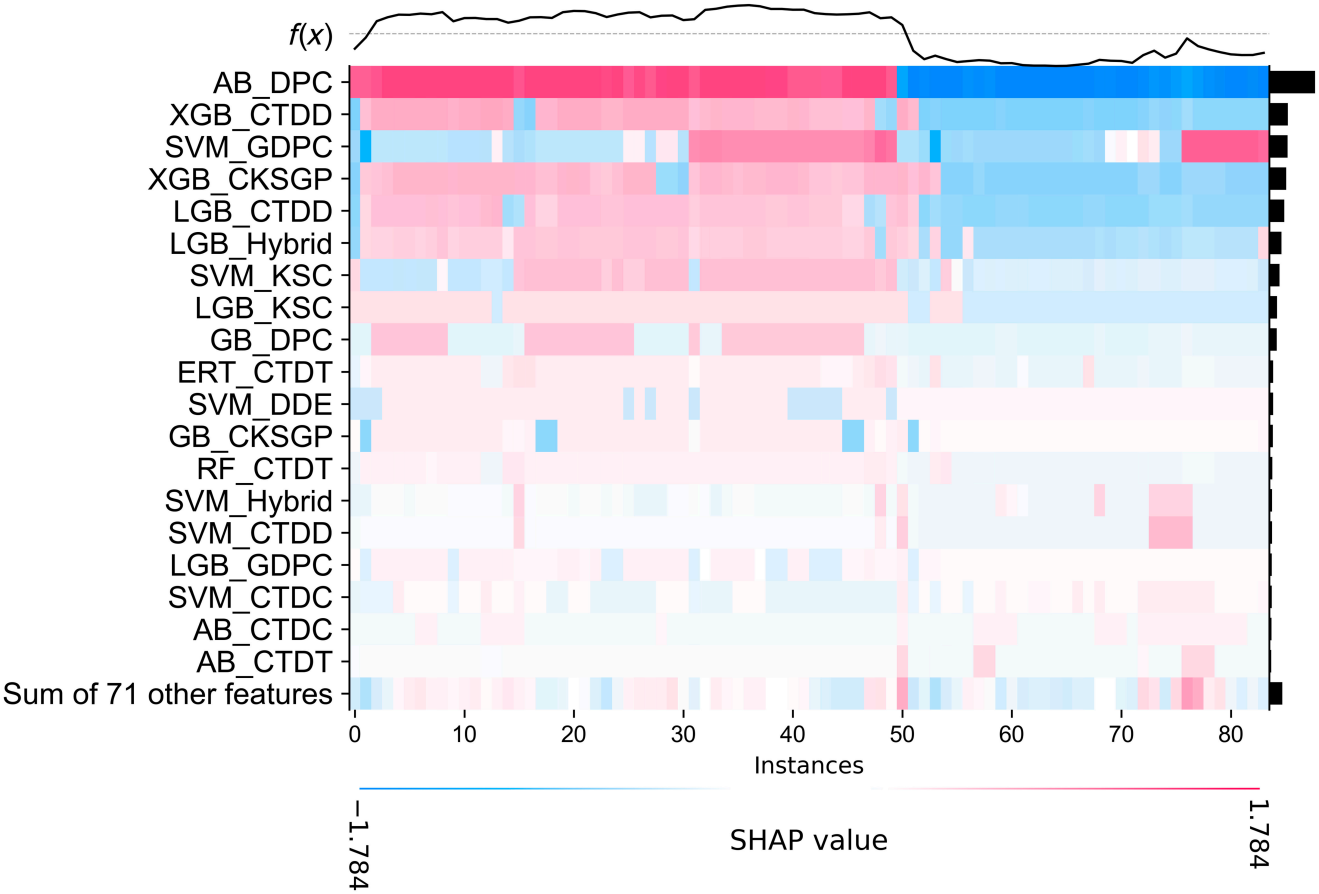
A heatmap plot of the SHAP values for the top 19 probabilistic features based on the training dataset.

The IML-TYLCVs were trained using the optimal class label feature vector that produced a better performance than the baseline predictors. However, there is a lack of information regarding the directionality and contribution of the class label features to the integrated model. The SHapley Additive Explanation (SHAP) has been used to illustrate the most significant features and their relationship to the results of IML-TYLCVs. Figure 6 shows that IML-TYLCVs generate predictions as line charts above the heatmap matrix (*f*(*x*)). Below the heatmap, bar graphs illustrate the global importance of each feature, and a list of the top 19 most important features is shown according to their global importance. The results indicate that 9 different encodings (DPC, CTDT, CTDC, CTDD, CKSGP, DDE, KSC, hybrid, and GDPC) based on 7 classifiers (RF, ERT, GB, SVM, AB, XGB, and LGB) contributed to the final prediction of IML-TYLCVs. Six SVM-based baseline models, 4 LGB-based baseline models, 3 AB-based baseline models, 2 XGB-based and 2 GB-based baseline models each, and 1 RF and 1 ERT-based baseline model each contributed the most to the final prediction. A higher value for most features is more likely to predict a severe strain, while a lower value is more likely to predict a mild strain. In conclusion, these results indicate that IML-TYLCV’s remarkable predictive performance can be attributed to both compositions and physicochemical properties (PCPs) derived from baseline models.

### Identification of novel TYLCVs

To collect and detect viral DNA occurrences in tomato samples, we processed through polymerase chain reaction (PCR) amplification using TYLCV-specific primers, all symptomatic samples were found positive as an amplicon of approximately 1.1 kb. In order to reduce external factor interference, we attempted to detect possible co-infection with tomato leaf curl New Delhi virus (ToLCNDV), tomato yellow leaf curl Thailand virus (TYLCTHV), and tomato yellow leaf curl Kanchanaburi virus (TYLCKaV). The PCR result showed that only TYLCV was detected, indicating there was no co-infection event in the collected samples. The coat protein (CP) of TYLCV amplicons were sequenced, and CP sequence analysis of 40 novel isolates using BLAST (basic local alignment search tool) showed 99.61% to 99.98% nucleotide sequence similarity with the TYLCV-isolated Gwangju30 (GenBank: HM856913) [24].

### Analysis of genetic variation and diversity of novel TYLCV groups

**Fig. 7.**
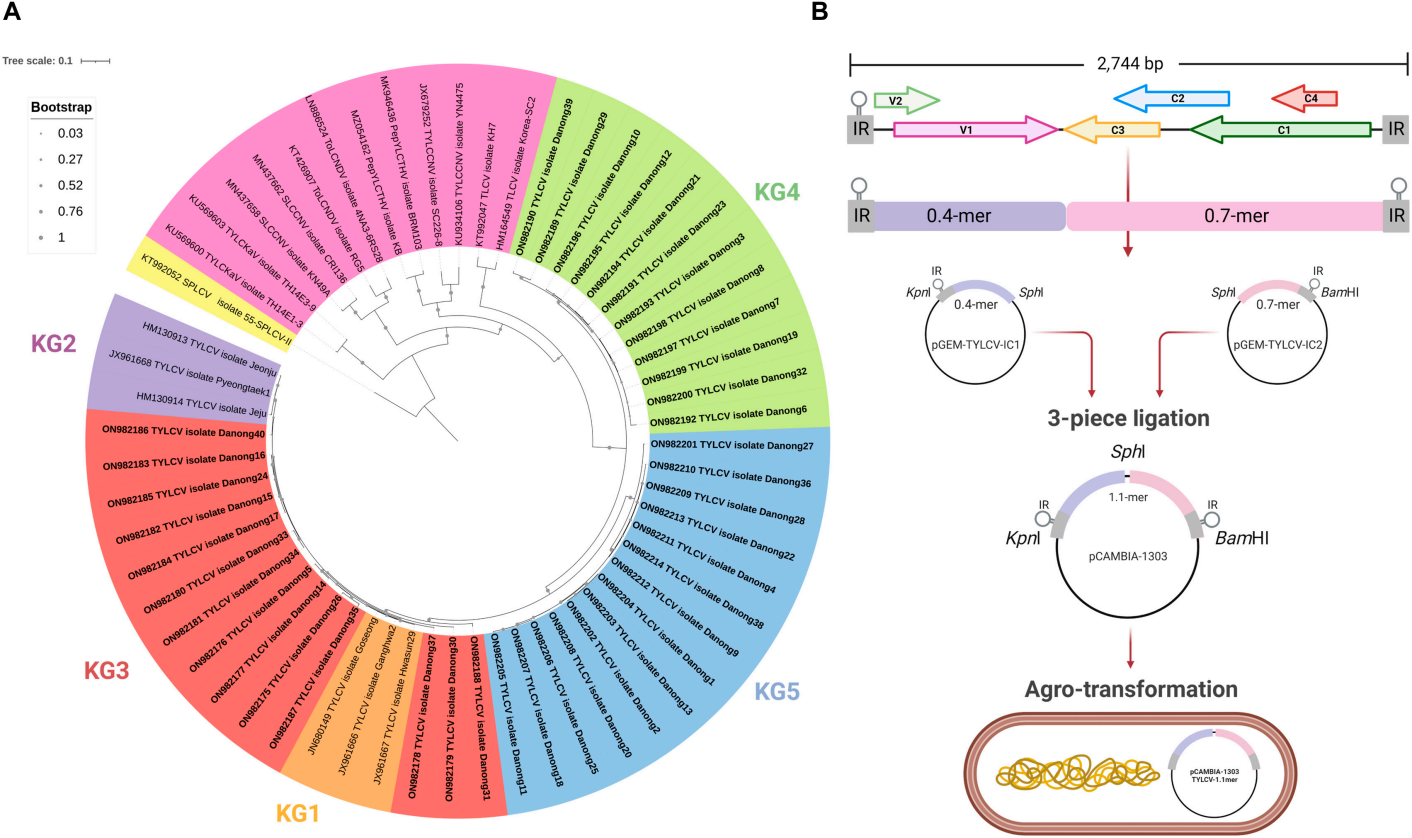
The results of the phylogenetic tree and the construction of infectious clones map of novel TYLCV isolates in this study. (A) The phylogenetic reconstruction of 40 samples of full-genome novel TYLCV isolates showed different clades; phylogenetic tree constructed using the maximum likelihood method at 1,000 bootstrap replicates in MEGA software version X. Additional full-genome sequences of begomovirues infecting tomatoes such as tomato yellow leaf curl Kanchanaburi virus (TYLCKaV), squash leaf curl China virus (SLCCNV), tomato leaf curl New Delhi virus (ToLCNDV), pepper yellow leaf curl Thailand virus (PepYLCTHV), tomato yellow leaf curl China virus (TYLCCNV), and tobacco leaf curl virus (TLCV) were obtained from NCBI GenBank. The full genome of the sweet potato leaf curl virus (SPLCV), which is a begomovirus noninfecting tomato, was used as an outgroup for the root tree. The colors in the phylogenetic tree represent the outgroup as the yellow clade, the other group as the pink clade, TYLCV-KG1 as the orange clade, TYLCV-KG2 as the purple clade, TYLCV-KG3 as the red clade, TYLCV-KG4 as the green clade, and TYLCV-KG5 as the blue clade. (B) The genome organization of TYLCV, DNA-A component consists of a conserved stem loop structure (hairpin) within the intergenic region (IR) and ORFs encoded on the virion sense (V) or complementary sense (C) strand. V1, coat protein; V2, movement protein; C1, replication-associated protein; C2, transcriptional activator protein; C3, replication enhancer protein; C4, pathogenicity protein. The TYLCV infectious clone construction used the partial tandem repeat construction. Two fragments of each component were generated, ligated with the pCAMBIA-1303 vector, and transformed into *Agrobacterium tumefaciens* strain GV3101.

The strain demarcation of the novel TYLCV groups was also identified. All 40 full-genome sequences had a pairwise identity of more than 91% with TYLCV isolated in Korea (GenBank: KF225312). Regarding the phylogenetic analysis, the results showed that 40 sequences of novel TYLCV isolates and TYLCV-KG1/KG2 were in separate clades, and 3 novel groups of TYLCV newly emerged in Korea. Fourteen isolates from the first group shared the most pairwise identity closely with TYLCV-KG1 referred to as TYLCV-KG3, 10 isolates known as TYLCV-KG4 were in the second group, and 16 isolates in the last group is classified as TYLCV-KG5 (Fig. 7A). The pairwise sequence alignment generated by Sequence Demarcation Tool (SDT) showed that 14 isolates of the TYLCV-KG3 group shared pairwise identity between 98.65% and 99.23% with TYLCV-KG1 (GenBank: HM130912), meanwhile 10 isolates of the TYLCV-KG4 group shared pairwise identity between 92.36% and 93.12% with TYLCV-KG2 (GenBank: HM130913), and 16 isolates of TYLCV-KG5 exhibited around 93% similarity with TYLCV-KG1 (GenBank: HM130912) (Fig. S5 and Table S2). The results indicate that TYLCV-KG3 is a variant of TYLCV-KG1, while TYLCV-KG4 and TYLCV-KG5 are new strains of TYLCV groups in Korea (KGs).

### Construction of infectious clones of novel TYLCV groups for virus challenging

**Fig. 8.**
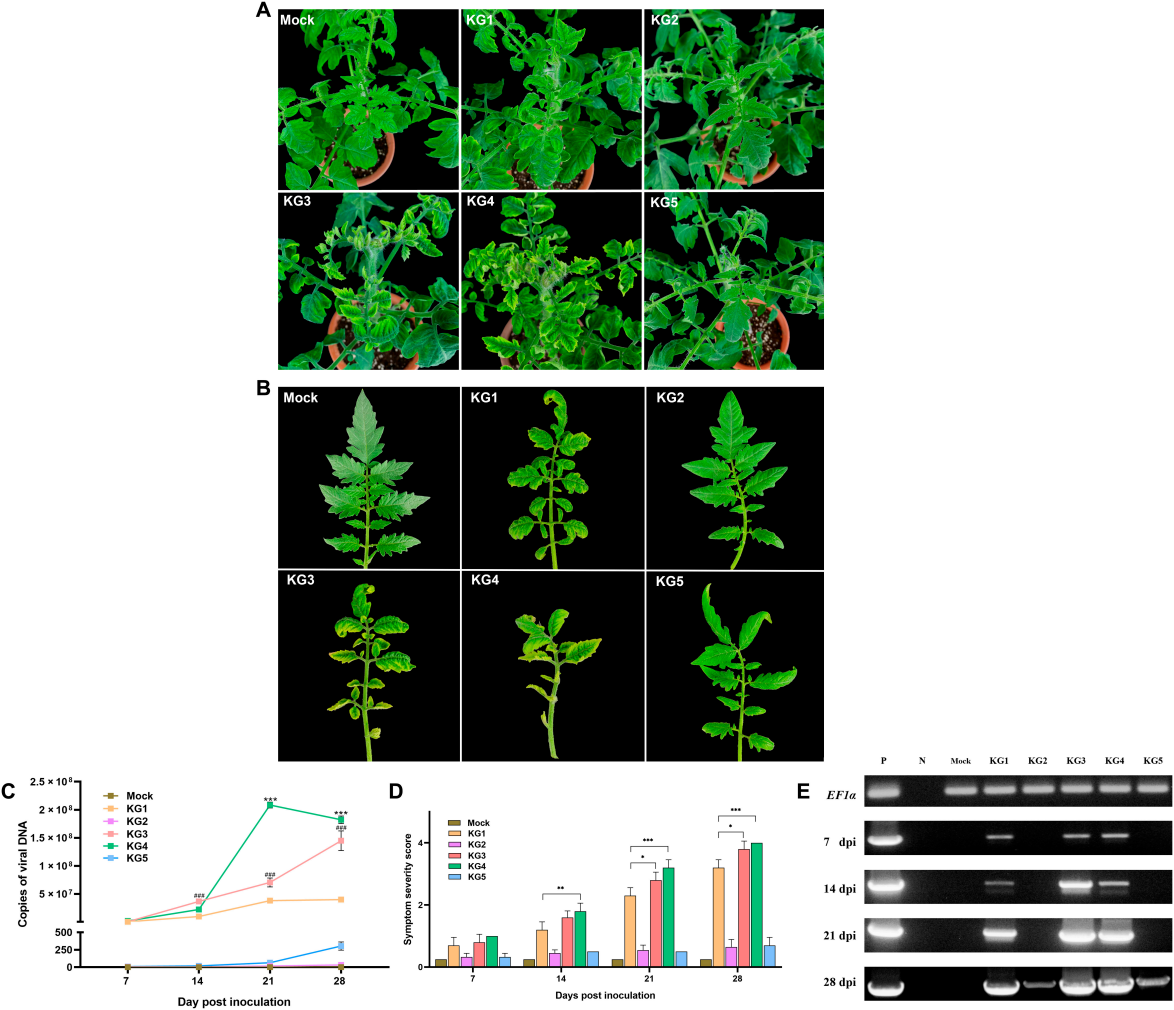
Plant phenotypes observation of agro-inoculation and quantitative results. The tomato plant phenotype observation of agro-inoculation with TYLCV novel group infectious clone including the quantitative viral DNA copy number using a standard curve and the symptom severity score. (A and B) Phenotypes observed in tomato cv. MoneyMaker plants at 28 dpi with infectious clones of TYLCV-KG1, TYLCV-KG2, TYLCV-KG3, TYLCV-KG4, TYLCV-KG5, and mock. (C and D) Average quantitative viral DNA copy number and average symptom severity score of the TYLCV 5 groups were assayed at 7 to 28 dpi. DNA copies were expressed in copies/μl. (E) PCR analysis with the leaf from 5 groups of TYLCV at 7 to 28 dpi on 1% agarose gel. Lane P, positive control; lane N, no template control; lane Mock, total DNA of mock plant control; lane KG1–5, total DNA from the leaf of 5 groups of TYLCV. (C): ^###^*P* < 0.001 for TYLCV-KG3 versus TYLCV-KG1, and ***P < 0.001 for TYLCV-KG4 versus TYLCV-KG3. (D): *P < 0.1 TYLCV-KG3 versus TYLCV-KG1, **P < 0.01 TYLCV-KG4 versus TYLCV-KG1, and ***P < 0.001 for TYLCV-KG4 versus TYLCV-KG1.

To confirm the blind prediction based on the development of plant phenotypes infected with the novel TYLCV groups, 3 TYLCV genomes were selected as TYLCV-KG3 (GenBank: ON982178), TYLCV-KG4 (GenBank: ON982198), and TYLCV-KG5 (GenBank: ON982202) groups as representatives of 40 TYLCV novel isolates. The infectious clones (Fig. 7B) of the 3 novel TYLCV isolates were constructed as pCAM1303-TYLCV-KG3, pCAM1303-TYLCV-KG4, and pCAM1303-TYLCV-KG5. Agro-inoculated tomato plants with 4 different infectious clones, except TYLCV-KG2, showed a very light yellowing of leaflet margins on apical leaves compared to mock plants at 7 day post inoculation (dpi). At 14 dpi, tomato plants that were inoculated with TYLCV-KG1, TYLCV-KG3, and TYLCV-KG4 clones showed phenotypes of yellowing and minor curling at the leaflet ends. After 21 dpi, leaves in inoculated plants exhibited inclusive leaf yellowing, curling, and a mild reduction of leaflet size depending on the infectious clones (Fig. S6). At 28 dpi, only TYLCV-KG3 and TYLCV-KG4 infected tomato plants developed symptoms of severe stunting, yellowing, major leaf cupping, and curling, whereas TYLCV-KG2 and TYLCV-KG5 infected plants showed milder symptoms (Fig. 8A and B). Mock-inoculated tomato plants with pCAMBIA-1303 as the control group did not produce any TYLCV symptoms. Strikingly, symptoms of TYLCV-KG3 and TYLCV-KG4 within 7 dpi are more severe compared to TYLCV-KG1, which was characterized as a severe strain [25].

### TYLCV symptom severity correlates with viral DNA copy numbers

To evaluate the plant phenotypes of the novel TYLCV groups based on TYLCV symptom severity score and viral DNA copy number, the inoculated plants were investigated. TYLCV symptom severity scores and TYLCV copy numbers in TYLCV-KG3 and TYLCV-KG4 infected plants increased gradually from 7 to 28 dpi. At 28 dpi, the symptom severity scores with copy numbers of TYLCV-KG1, TYLCV-KG2, TYLCV-KG3, TYLCV-KG4, and TYLCV-KG5 were 3.2 ± 0.2 with 4.01 ± 1.12 × 10^7^ copy numbers/μl, 0.7 ± 0.2 with 31.83 ± 3.49 copy numbers/μl, 3.8 ± 0.2 with 1.46 ± 0.15 × 10^8^ copy numbers/μl, 4.0 ± 0.0 with 1.82 ± 0.64 × 10^8^ copy numbers/μl, and 0.7 ± 0.2 with 1.82 ± 0.64 × 10^8^ copy numbers/μl, respectively (Table S3). Compared to TYLCV-KG1, known as TYLCV severe strain [13] in 2010, TYLCV-KG3 and TYLCV-KG4 showed significantly more severe symptoms at 14, 21, and 28 dpi (Fig. 8C and D). The infectivity of TYLCV was further confirmed by PCR using TYLCV detection primers (Fig. 8E).

### The novel TYLCV group breaking down TYLCV resistance phenotype in *Ty*-1 and *Ty*-2 breeding line

**Fig. 9.**
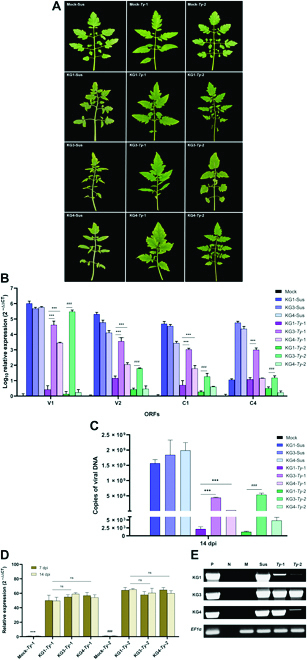
Phenotype observation of tomato breeding lines of agro-inoculation with the novel severe strains, their relative gene expression, and quantitative viral DNA copy results. (A) Phenotypes observed in tomato breeding lines consist of *Ty*-1, *Ty*-2, and susceptible line at 14 dpi with infectious clones of TYLCV-KG1, TYLCV-KG3, TYLCV-KG4, and mock. (B) The log_10_ relative fold ORF-gene expression levels of TYLCV-KG1, TYLCV-KG3, and TYLCV-KG4 challenging with tomato breeding lines; we calibrated the values with mock plant levels (set to 1) and normalized relative to *EF1α* gene. (C) Relative fold in gene expression of the *Ty*-1 and *Ty*-2 gene against TYLCV-KG1, TYLCV-KG3, and TYLCV-KG4. The values were normalized and calibrated with mock *Ty*-1 and *Ty*-2 plant levels at 7 to 14 dpi (set to 1). (D) Average quantitative viral DNA copy number of TYLCV-KG1, TYLCV-KG3, and TYLCV-KG4 challenging with tomato breeding lines assayed at 14 dpi. (E) PCR analysis using the leaf from inoculated breeding lines (*Ty*-1, *Ty*-2, and susceptible line) challenged with TYLCV-KG1, TYLCV-KG3, and TYLCV-KG4 at 14 dpi on 1% agarose gel. Lane P, positive control; lane N, no template control; lane Mock, total DNA of mock plant control; lane Sus, susceptible line; lane *Ty*-1, *Ty*-1 breeding line; lane *Ty*-2, *Ty*-2 breeding line; KG1–4, total DNA from the inoculated leaf of 3 groups of TYLCV. DNA copies were expressed in copies/μl. (B) and (C): ***P < 0.001 for TYLCV-KG3 or TYLCV-KG4 versus TYLCV-KG1 in *Ty*-1 breeding lines and ^###^*P* < 0.001 for TYLCV-KG3 versus TYLCV-KG1 in *Ty*-2 breeding lines with different four ORFs. (D): ***P < 0.001 and ^###^*P* < 0.001 for mock breeding lines versus inoculated breeding lines and ns for not significant.

To further evaluate the symptom severity of the novel TYLCV groups using TYLCV resistance tomato breeding lines harboring *Ty*-1 or *Ty*-2, tomato breeding lines were inoculated with the infectious clones of 3 severe strains (TYLCV-KG1, TYLCV-KG3, and TYLCV-KG4). At 7 dpi, *Ty*-1 and *Ty*-2 breeding lines inoculated with TYLCV-KG1, TYLCV-KG3, and TYLCV-KG4 started to show minor leaf curling. After 14 dpi, both TYLCV-KG3-inoculated *Ty*-1 and *Ty*-2 breeding lines displayed leaf yellowing and curling; meanwhile, only the TYLCV-KG4-inoculated *Ty*-1 breeding line continued to show TYLCV symptoms (Fig. 9A). However, in tomato plants that acquired *Ty*-1 and *Ty*-2, inoculated with TYLCV-KG1 showed very mild symptoms in comparison with mock plants. Additionally, at 14 dpi, the relative expression of 4 ORFs (V1, V2, C1, and C4) in TYLCV-KG3 was significantly expressed on *Ty*-1 and *Ty*-2 breeding lines comparing with TYLCV-KG4 (*P* < 0.001). On the contrary, TYLCV-KG4 only showed their substantial expression on Ty-1 breeding lines compared to TYLCV-KG1 (*P* < 0.001) (Fig. 9B). Results of viral copy numbers of TYLCV-KG1, TYLCV-KG3, and TYLCV-KG4 in tomato breeding lines at 14 dpi were shown in Fig. 9C and Table S4. The viral copy numbers in TYLCV-KG3 on *Ty*-1 and *Ty*-2 breeding lines was significantly higher (*P* < 0.001); on the other hand, TYLCV-KG4 was significantly higher (*P* < 0.001) only on *Ty*-1 breeding lines compared with TYLCV-KG1. However, the relative gene expression of the *Ty*-1 and *Ty*-2 genes at 2 time points on 7 and 14 dpi after novel severe TYLCV-inoculated plants was significantly higher (*P* > 0.001) than mock-inoculated plants, and no significant difference was observed in their expression levels among TYLCV-inoculated plants at any time point (Fig. 9D). The infectivity of TYLCV in tomato breeding lines was confirmed by PCR in the second week after inoculation (Fig. 9E). As a result, TYLCV-KG3 is the most severe isolate compared with all treatments using symptoms of TYLCV severity and viral copy numbers.

## Discussion

ML has recently been applied to a variety of fields, including life sciences and mobile applications used on a daily basis. Bioinformatics researchers develop prediction models on the basis of experimental data, with the assumption that these models will be useful to experimentalists. As a result, many prediction models have been developed [25], but their efficiency cannot be evaluated in real time, resulting in a huge gap between method developers and experimental scientists. Computational biologists, bioinformaticians, and computer scientists collaborated closely with experimentalists during the recent COVID-19 pandemic to assist medical experts and policymakers [26], ultimately saving the lives of many people. In this study, we proposed a novel integrated ML framework called IML-TYLCVs that accurately predicts mild/severe strains from the sequence information. The IML-TYLCVs makes use of 90 baseline models that have been trained using 12 different feature encodings and 8 different classifiers.

In sequence analysis, 2 novel groups of TYLCV strains (TYLCV-KG4 and TYLCV-KG5) were found to share approximately 92% and 93% sequence identity to TYLCV-KG2 and TYLCV-KG1, respectively [31]. However, TYLCV-KG3 shared a pairwise identity of 98% with TYLCV-KG1. On the basis of the phylogenetic and sequence analysis, it is hard to reveal the novel gene function. In general, phylogenetic analyses were carried out using DNA sequences to predict the relationships among species [27]. However, there are studies where gene functions could not be determined through phylogenetic analysis [28] even though they used high-throughput gene expression data [29]. Interestingly, IML-TYLCVs accurately predict the symptom severity of novel groups using ORF information. However, when we developed the prediction models based on genome sequences, the preliminary results showed 100% accuracy on the training dataset, and blind prediction showed that TYLCV-KG4 and TYLCV-KG5 were severe strains and TYLCV-KG3 was a mild strain. This is mainly due to the bias in the genome sequence. To overcome such bias, we used ORFs (protein sequences) to develop a reliable prediction model.

To verify and confirm the computational predictions, tomato plants were inoculated with infectious clones and obtained from 5 different groups. Among these, TYLCV-KG4 was observed to be the most severe strain based on tomato plant phenotypes, symptom severity scores, and viral DNA copy number. However, TYLCV-KG2 and TYLCV-KG5 produced mild symptoms. In the case of tomato breeding lines, TYLCV-KG3 was the most severe strain compared to the other strains. In previous studies, 2 strains of TYLCV (KG1 and KG2) did not induce any symptoms in TYLCV-resistant cultivars [10]. We expect that these 2 severe TYLCV strains (KG3 and KG4) might be mutated to become more infectious in tomatoes harboring *Ty* genes. Although TYLCV-KG3 has a high sequence identity to TYLCV-KG1, TYLCV-KG3 shows more severe symptoms mainly because of a few mutations hidden in the DNA sequence information. Another reason might be ecological fitness as a mechanism for increasing their evolutionary potential and local adaptation [30]. In general, the *Ty-1* gene has been the major focus in TYLCV resistance worldwide [31,32]. However, *Ty-1*-resistant tomato has been observed as not effective in the fields and against mixed infection [33]. Therefore, we collected 40 tomatoes with typical TYLCV-like symptoms from TYLCV-resistant cultivars harboring *Ty* genes and made sure that there was no mixed infection.

Further research is needed to understand the roles and functions of mutated genes and altered nucleotide sequences among different groups of KGs. With a large number of DNA sequences of different KGs available in the future, we plan to apply ML-based novel approaches directly identify mutations that alter the function of KGs, which will assist experimentalists in annotating uncharacterized sequences. On the basis of the limited resources available at the moment, we developed an integrated computational framework for identifying novel functions for KGs and then validating these functions experimentally. Nevertheless, the present study has the following limitations: (a) a smaller training dataset due to a lack of properly updated sequences. (b) Prediction model developed exclusively on TYLCVs that may not apply to other species, including begomoviruses. For the convenience of experimentalists, we have provided the user-friendly online IML-TYLCVs web server that can be accessed at https://balalab-skku.org/IML-TYLCVs. Plant virologists and plant breeding professionals can use our web server to obtain information that will assist them in developing more effective strategies for combating newly emerging viruses.

## Materials and Methods

### Dataset construction

The nucleotide sequences TYLCV-KG1 and TYLCV-KG2 (Table S1) are considered as the training dataset. However, uncharacterized sequences TYLCV-KG3, TYLCV-KG4, and TYLCV-KG5 (from Korea) were used for blind prediction. The ORF finder tool [34] was used to convert all nucleotide sequences into ORFs (protein sequences), including V1, V2, C1, C2, C3, and C4 proteins. Consequently, we excluded identical ORFs that resulted in 50 TYLCV-KG1, 34 TYLCV-KG2, 52 TYLCV-KG3, 42 TYLCV-KG4, and 57 TYLCV-KG5 sequences. In particular, TYLCV-KG1 and -KG2 are used for the development of prediction models, while the remaining sequences are used for blind prediction.

### Feature encodings

In this study, we employed 11 different feature encodings (AAC, CTDT, GTPC, CKSGP, DDE, KSC, CTDC, DPC, QSO, CTDD, and GDPC) and hybrid features (linear combination of the 11 encodings). The mathematical expressions of these encodings have been discussed in previous studies [35–37], and they can be extracted using BioSeq-Analysis [38] and iFeature [39]. The following is a brief description of these encodings.

#### AAC

It encodes 20D features describing the proportion of each standard amino acid residue present in a specific protein sequence.

#### DPC

Another commonly used encoding for protein/peptide-based classification. DPC encodes 400D features that provide information about all possible DPCs present in the given sequence.

#### GDPC and GTPC

The standard 20 amino acids can be classified into 5 groups according to their PCPs: aromatic (g1 ∈ F, Y, and W), positively charged (g2 ∈ R, K, and H), aliphatic (g3 ∈ A, V, G, L, M, and I), uncharged (g4 ∈ C, P, N, S, T, and Q), and negatively charged (g5 ∈ D or E). Using these properties, GTPC and GDPC generate 125D and 25D features, respectively.

#### CTDC, CTDT, and CTDD

The PCPs (solvent accessibility, normalized van der Waals volume, polarizability, hydrophobicity, charge, polarity, and secondary structures) have been classified into 3 groups. These properties enable CTDC, CTDD, and CTDT to encode 39D, 195D, and 39D features, respectively.

#### KSC

The KSC descriptor is derived from the Conjoint CTriad descriptor, which calculates both the number of three continuous amino acid units and continuous amino acid units separated by any *k* residues (*k* = 5). The KSC encodes 343D feature vectors.

#### QSO

The QSO takes into account of sequence order effect (i.e., PCP distance between amino acids) and generates a 100D feature vector.

#### CKSGP

A CKSGP is a variation of the composition of *k*-spaced amino acid pair descriptor that computes the frequency of amino acid group pairs separated by any *k* residues (*k* = 10). Finally, CKSGP encodes the 275D feature vector.

#### DDE

Three parameters are computed to construct the DDE feature vector: the theoretical mean (*T*_m_), DPC, and theoretical variance (*T_γ_*).

$$\left\{\begin{array}{c} \mathrm{DPC}=\frac{f_{\mathrm{pq}}}{K-1},p,q\in \left\{A,C,D,E,F,\ldots ,Y\right\}\\ T_{m}=\frac{C_{p}}{C_{N}}\times \frac{C_{q}}{C_{N}}\\ T_{\gamma }=\frac{T_{m}\left(p,q\right)\left(1-T_{m}\left(p,q\right)\right)}{K-1}\\ \mathrm{DDE}=\frac{\mathrm{DPC}-T_{m}}{\sqrt{T_{\gamma }}} \end{array}\right.$$ (1)

where *C_p_* and *C_q_* are the number of codons that code for the first and the second amino acids, respectively, in the given peptide “*ab*”. *The C_N_* was 61, excluding stop codons.

#### Hybrid

It is a linear combination of all 11 encodings, which resulted in a 1961D feature vector.

### Conventional ML algorithms

We considered 8 different ML classifiers: SVM, RF, ERT, GB, AB, XGB, LGB, and ANN. Details of these algorithms and parameter ranges have been provided in our previous studies [40,41]. Here, we have employed the same parameter search ranges for tuning each of the ML hyperparameters by running 50 times randomized 10-fold cross-validation. A median parameter was used in the development of the respective ML-based final prediction model. Notably, we employed all possible commonly used classifiers for systematic analysis to develop a novel integrated framework.

### Model evaluation

The 5 commonly used evaluation metrics were considered to evaluate the model performance [42,43], including MCC, Sn, Sp, ACC, and AUC. The metrics are defined as follows:

$$\left\{\begin{array}{c} \mathrm{Sn}=\frac{\mathrm{TP}}{\mathrm{TP}+\mathrm{FN}}\\ \mathrm{Sp}=\frac{\mathrm{TN}}{\mathrm{TN}+\mathrm{FP}}\\ \mathrm{ACC}=\frac{\mathrm{TP}+\mathrm{TN}}{\mathrm{TP}+\mathrm{TN}+\mathrm{FN}+\mathrm{FP}}\\ \mathrm{MCC}=\frac{\mathrm{TP}\times \mathrm{TN}-\mathrm{FP}\times \mathrm{FN}}{\sqrt{\left(\mathrm{TP}+\mathrm{FN}\right)\left(\mathrm{TP}+\mathrm{FP}\right)\left(\mathrm{TN}+\mathrm{FP}\right)\left(\mathrm{TN}+\mathrm{FN}\right)}} \end{array}\right.$$ (2)

where TP, TN, FP, and FN denote the true positives, true negatives, false positives, and false negatives, respectively. Furthermore, receiver operating characteristic curves and AUC values were used to assess the overall performance.

### DNA extraction of the novel TYLCVs

The leaves of tomato plants (*Solanum lycopersicum*) showing the typical symptoms of TYLCV disease such as curling, yellowing, and stunted growth were collected from different farms located in Korea in November 2021. Forty isolated TYLCV samples from 40 Danong tomatoes were collected from Chungcheongnam-do, Ganwon-do, Gwangju, Gyeongnam-do, and Jeollanam-do (Table S5 and Fig. S7). Using the Viral Gene-spin Viral DNA/RNA Extraction Kit (iNtRON Biotechnology, Seongnam, Korea), viral DNA was extracted from 40 samples from various locations.

### Viral DNA detection and genome cloning

Viral DNA detection was performed using the T100 Thermal Cycler (Bio-Rad, Hercules, CA, USA) with a final reaction volume of 20 μl, which contains TYLCV-specific primers encoding the V1 gene of TYLCV isolates based on a previous study [13]. The specific primers of 3 species of Begomovirus-infecting tomato, including ToLCNDV, TYLCTHV, and TYLCKaV, were designed for the detection of newly emerging viruses and diseases using Primer-BLAST (Table S6) [44] and a universal primer pair specific for alphasatellite and betasatellite [45,46], to test whether DNA satellites were associated with these isolates. The AccuPower ProFi Taq PCR Master Mix (Bioneer, Daejeon, Korea) was used for the amplification and DNA detection in PCR. The PCR conditions were as follows: an initial denaturation at 94 °C for 3 min, followed by 35 cycles (denaturation at 94 °C for 30 s, annealing at 58 °C for 30 s, and extension at 72 °C for 1 min), and a final extension at 72 °C for 10 min. Then, the PCR products were electrophoresed on a 1% agarose gel. Furthermore, the PCR products were sequenced using Sanger sequencing at the Macrogen Institute (Macrogen, Seoul, Korea). In this experiment, the PCR reaction per DNA sample was performed at least 3 times.

Newly designed full-genome primers were used to amplify the full sequence of TYLCV (Table S6). The target viral DNA was cloned into the pGEM T-easy vector (Promega, Madison, USA) and then individual recombinant plasmids were sequenced at the Macrogen Institute (Macrogen, Seoul, Korea) and submitted to GenBank. After that, the obtained sequences were compared to their identities using the BLAST program (http://blast.ncbi.nlm.nih.gov/Blast.cgi). The full genomes of 40 isolated TYLCV samples in this study were submitted to GenBank and assigned accession numbers (Table S2).

### Sequence analysis and pairwise comparisons

To organize and distinguish the evolutionary phylogenetic relationships of TYLCV in Korea, 2 datasets were assembled and aligned with MUSCLE [47]. First, a pairwise sequence alignment was performed on the TYLCV dataset and generates identities between every pair of sequences in the dataset with SDT software version 1.2 (http://www.cbio.uct.ac.za/SDT) as recommended by the International Committee on Taxonomy of Viruses *Geminiviridae* study group. Second, the phylogenetic reconstruction of 40 samples of novel TYLCV isolates and the full-genome sequences of the reported TYLCV in Korea (KG1 and KG2) was constructed using the maximum likelihood criterion at 1,000 bootstrap replicates in MEGA software version X [48]. Additional whole DNA-A genome sequences of tomato-infecting begomoviruses and the sweet potato leaf curl virus (SPLCV) as an outgroup (tomato-noninfecting begomoviruses) were obtained from NCBI GenBank (Table S7) during the phylogenetic analysis.

### Construction of TYLCV infectious clone and agro-inoculation

The novel isolated infectious clones of TYLCV were constructed using a partial tandem repeat of the full-genome viral DNA method, as described previously [49]. Two fragments (IC1-0.4-mer and IC2-0.7-mer) of partial tandems were amplified from the full-length recombinant plasmid of novel TYLCV isolates using 2 primer sets (TYLCV-IC1-F/TYLCV-IC1-R and TYLCV-IC2-F/TYLCV-IC2-R) (Table S8) for each group. Each fragment was ligated into a pGEM-T Easy Vector (Promega, Madison, USA) to generate pGEM-TYLCV-IC1 and pGEM-TYLCV-IC2. The cloned fragments were digested with *Kpn*I, *Sph*I, and *Bam*HI and cloned into digested pCAMBIA-1303 (pCAM1303TYLCV-1.1mer) (Fig. 7B). The infectious constructs of the novel TYLCV groups were transformed into *A. tumefaciens* strain GV3101 using the freeze-thaw transformation method.

Tomatoes cultivar cv. Money-makers (TYLCV-susceptible) were planted in a walk-in growth chamber at Sungkyunkwan University (Korea) and five of 4-week-old plants per replication (total of 15 plants/treatment) were inoculated with *A. tumefaciens* strain GV3101 containing pCAMBIA-1303 TYLCV-KG1/KG2-infectious clones [49] as positive controls and TYLCV-KG3, TYLCV-KG4, and TYLCV-KG5 infectious clones in the present study. The TYLCV-resistant tomato breeding line harbors *Ty*-1, *Ty*-2, and susceptible cultivars that were inoculated with the 3 severe strains (TYLCV-KG1, TYLCV-KG3, and TYLCV-KG4). Mock plants and TYLCV-susceptible lines were used as negative and positive controls, respectively. Cell cultures of each clone were grown at 28 °C in Luria-Bertani broth with rifampicin, gentamycin, and kanamycin until the optical density at 600 nm reached 1.0 and then inoculated on the apical side of plants using needles and plastic dropping pipette [50]. All inoculated plants were kept in a plant growth room with a photosynthesis period of 16 h of light and an air temperature of 28/22 °C (day/night).

### Phenotype observation and PCR analysis

The phenotypes of plants were observed weekly after inoculation for the TYLCV symptoms. Severity scoring was performed using a 4-point TYLCV symptom severity score according to Friedmann et al. [51], and TYLCV-infected genomic DNA was isolated using the FavorPrep Plant Genomic DNA Extraction Mini Kit (Favorgen, Ping-Tung, Taiwan). Then, the PCR results were detected with TYLCV-det-F/R under the same PCR conditions for each plant every week after inoculation.

### Viral copy number analysis

To quantify the TYLCV DNA after performing the analyzed relationship between viral DNA copy number and symptom development, we use a plasmid of pCAMBIA-1303 TYLCV-KG1 as a standard in accordance with the absolute quantity standard curve [52] (*Y* = −3.36*X* + 37.75, slope = −3.36, intercept = 37.75, amplification efficiency = 0.98, and *R*^2^ = 0.998). The extracted TYLCV DNA from the infected tomato leaves in the agro-inoculation experiment was quantified by obtaining DNA copies in 1 μl and calculating the average TYLCV DNA copies (copy number/μl) per sample. The TYLCV genomic DNA isolation from leaves of TYLCV-infected tomato plants was tested by real-time quantitative PCR (qPCR) every week after the inoculation of 5 different TYLCV groups. Reactions were performed using the SYBR premix Ex Taq (TaKaRa, Otsu, Japan) with specific primer sets based on the sequence of the V1 coding gene (nucleotides 883 to 1085), and the elongation factor 1α (*EF1α*) gene was measured in parallel as an endogenous control (Table S6). qPCR was performed using a Rotor Gene Q thermocycler (QIAGEN, Hilden, Germany), consisting of predenaturation at 95 °C for 5 min, followed by 40 cycles of denaturation at 95 °C for 10 min, annealing at 60 °C for 20 s, and extension at 72 °C for 20 s. The annealing temperature was selected according to the melting temperature of each primer.

### ORF-TYLCV, *Ty*-1, and *Ty*-2 gene expression analysis

To analyze gene expression, 25 ng of purified total RNA of all tomato breeding lines was extracted using the RNeasy Plant Mini kit (QIAGEN, Hilden, Germany) and treated with deoxyribonuclease following the manufacturer’s instructions. Then, complementary DNA (cDNA) was synthesized using the CellScript All-in-One cDNA Master Mix (CellSafe, Yongin, Korea) according to the protocol of the manufacturer. Real-time qPCR was performed in a Rotor Gene Q thermocycler (QIAGEN, Hilden, Germany) using similar conditions applied for the viral copy number analysis. The expression levels of TYLCV-ORF, *Ty*-1 [53], and *Ty*-2 [54] genes were determined by using specific primers (Table S9). An endogenous control (*EF1α*) was used to measure gene expression. The relative gene expression was calculated according to the following formula: 2^−∆∆CT^ [55].

### Statistical analysis

In each experimental process, 3 biological replicates were used, and each experimental treatment was repeated 3 times. The results are presented as either mean ± SD or error bars. One-way or two-way analysis of variance (ANOVA) was applied to compare statistical differences between the experimental groups. Statistical significance was defined as a *P* value of <0.05.

## Data Availability

Data are available from the authors upon reasonable request.
