# Supplementary material for: An Effective Integrated Machine Learning Framework for Identifying Severity of Tomato Yellow Leaf Curl Virus and Their Experimental Validation
Source: Research (Wash D C). 2023 Jan 10;6:0016. doi: 10.34133/research.0016 (PMC10013792; doi:10.34133/research.0016)
Supplement: Supplementary Materials — Figs. S1 to S7 Tables S1 to S9 [file research.0016.f1.docx]

**An effective integrated machine learning framework for identifying severity of Tomato yellow leaf curl virus and their experimental validation**

Nattanong Bupi^1^, Vinoth Kumar Sangaraju^2^, Le Thi Phan^2^, Aamir Lal^1^, Thuy Thi Bich Vo^1^, Phuong Thi Ho^1^, Amir Qureshi^1^, Marjia Tabassum^1^, Sukchan Lee^1,^ * and Balachandran Manavalan^2,^ *

^1^Department of Integrative Biotechnology, College of Biotechnology and Bioengineering, Sungkyunkwan University, Suwon 16419, Gyeonggi-do, Republic of Korea.

^2^Computational Biology and Bioinformatics Laboratory, Department of Integrative Biotechnology, College of Biotechnology and Bioengineering, Sungkyunkwan University, Suwon 16419, Gyeonggi-do, Republic of Korea.

*Corresponding authors

Sukchan Lee; [cell4u@skku.edu](mailto:cell4u@skku.edu) and Balachandran Manavalan; [bala2022@skku.edu](mailto:bala2022@skku.edu)

**
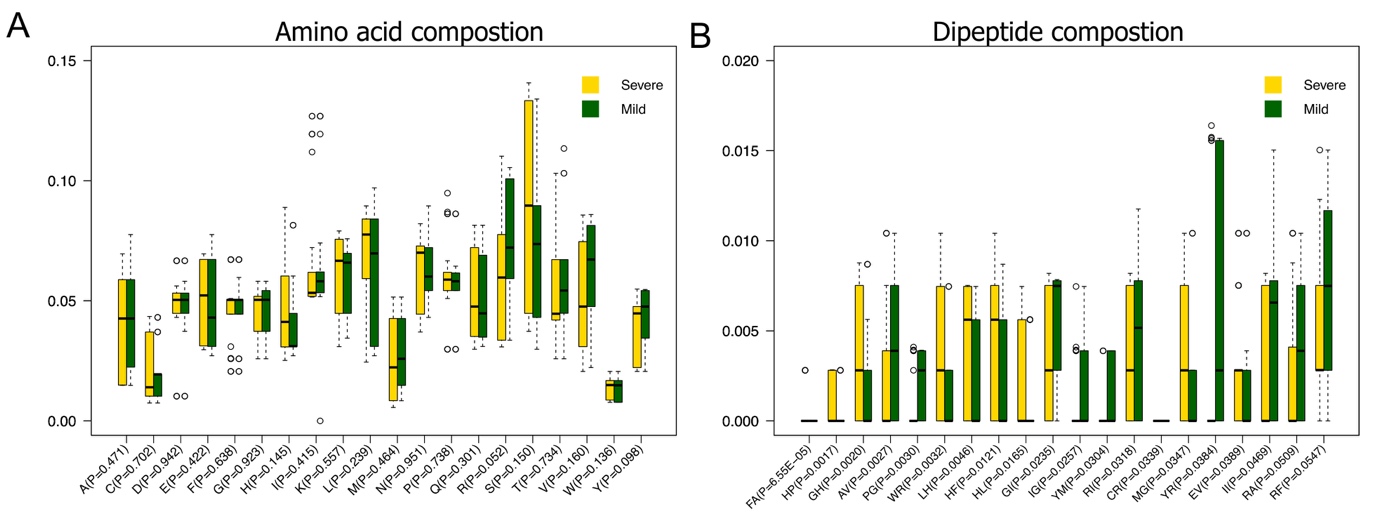
**

**Figure S1.** (A) Boxplots of normalized amino acid compositions of 20 amino acids for severe and mild strains based on the training dataset. X- and Y-axes represent 20 amino acids along with their *p*-value. (B) Boxplots of normalized dipeptide composition of top 20 dipeptides shown based on their *p*-value.

**Figure S2.** Performance comparison of different classifiers based on probabilistic features with different dimension. (A) random forest (RF), (B) gradient boosting (GB), (C) extremely randomized tree (ERT), (D) light gradient boosting (LGB), (E) extreme gradient boosting (XGB), (F) Adaboost (AB), (G) support vector machine (SVM), and (H) multi-layer perceptron (MLP).

**Figure S3.** Performance comparison of different classifiers based on class label features with different dimension.

**Figure S4.** Performance comparison of different classifiers based on PC (a combination of probability and class) features with different dimension.


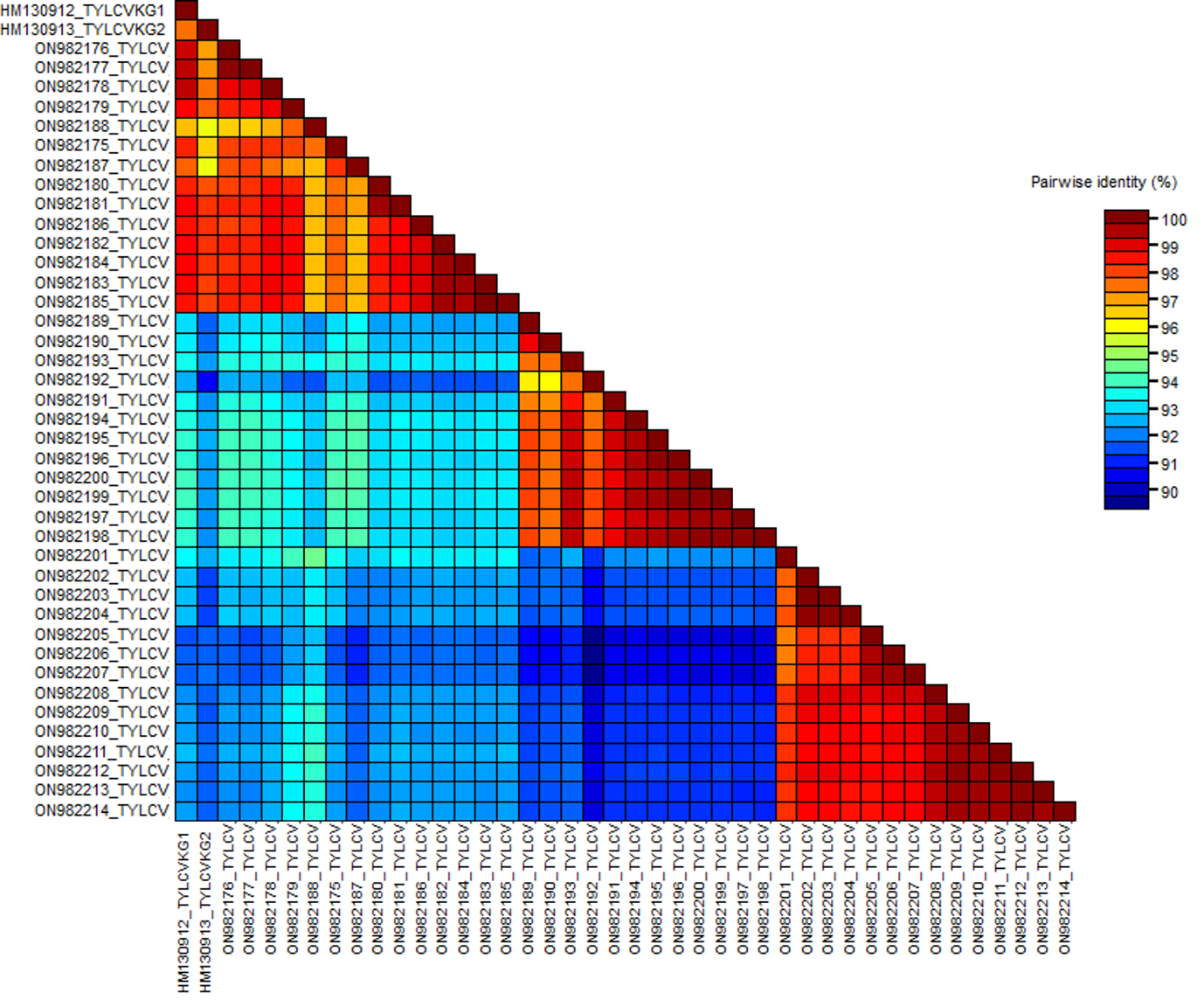


**Figure S5.** The colour coded matrix of pairwise similarity scores of 40 TYLCV new isolates with TYLCV-KG1 and KG2 by Sequence Demarcation Tool (SDT) software version 1.2.


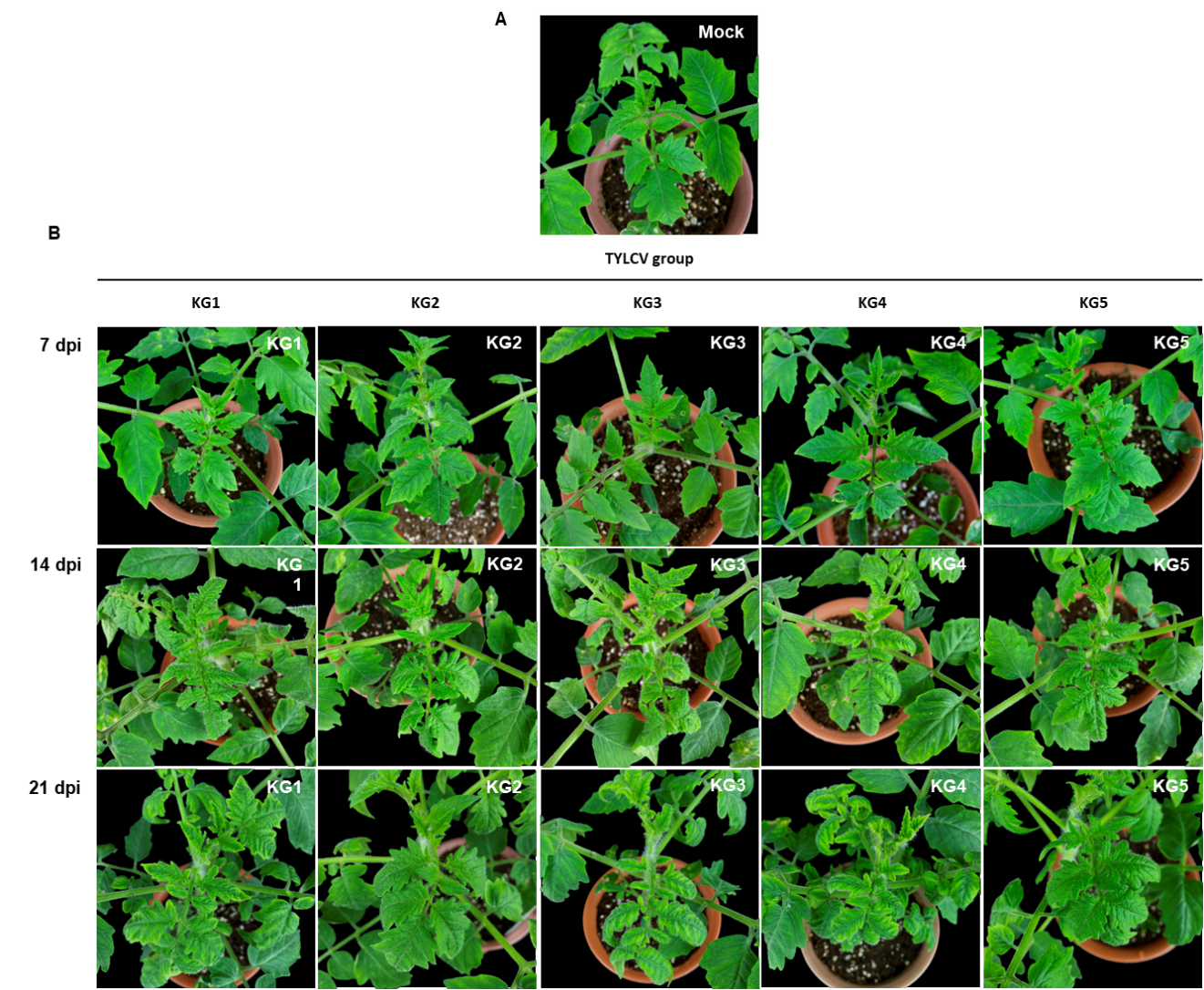


**Figure S6.** Phenotypes of tomato challenging with TYLCV-KG1, 2, 3, 4 and 5. At 7 dpi, at 14 dpi and at 21 dpi (B) compared with mock plants (A).

**
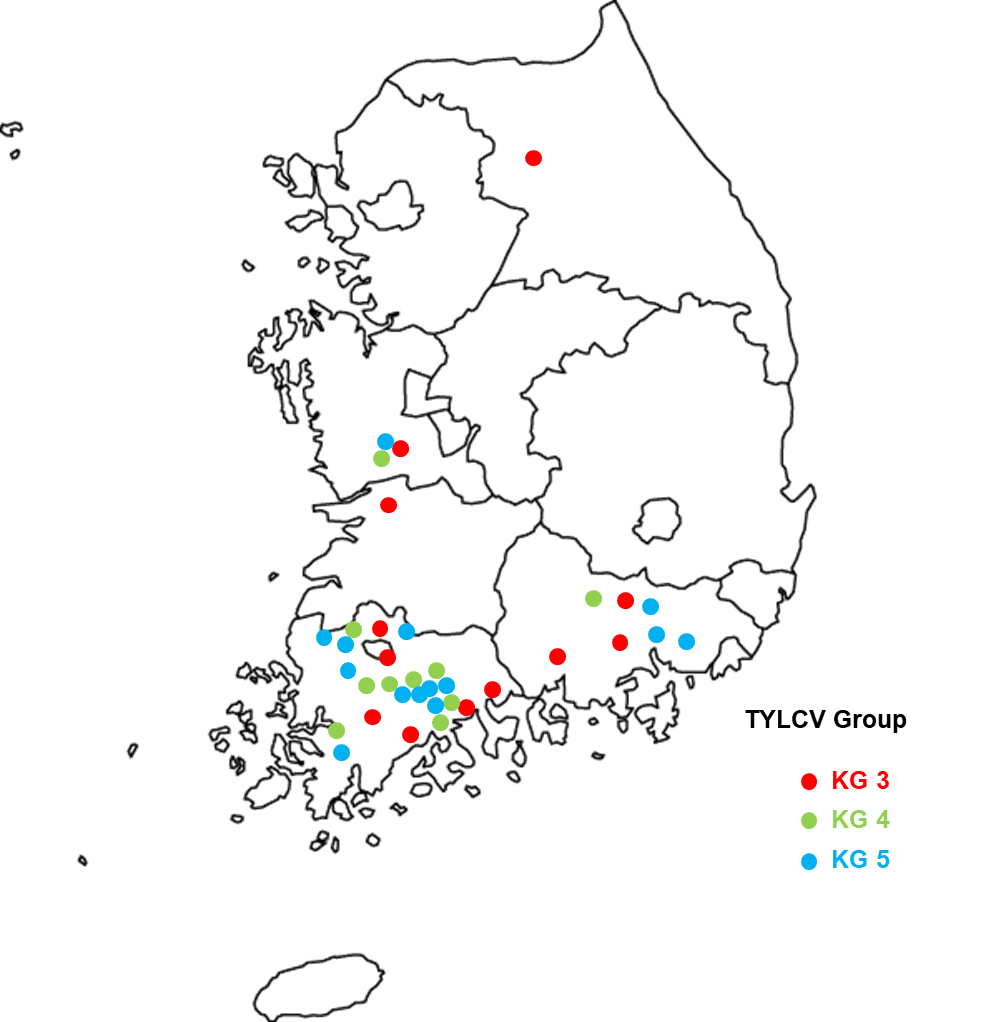
**

**Figure S7.** Location of regions where the novel TYLCV occurs and sample collection sites in Korea.

**Table S1.** The list of TYLCV sequences used for dataset construction.

| **GenBank no.** | **Isolate** | **GenBank no.** | **Isolate** |
| --- | --- | --- | --- |
| HM856919 | TYLCV-Uiseong41 | AB669434.1 | TYLCV-CC |
| HM856915 | TYLCV-Gyeongju52 | AB636412.1 | TYLCV-CJ-Kdo |
| HM856912 | TYLCV-Gunwi19 | AB636410.1 | TYLCV-CJ-cgh |
| HM130912 | TYLCV-Masan | AB636264.1 | TYLCV-YS |
| HM856911 | TYLCV-Goseong21 | GU325633.1 | TYLCV-Jeju |
| HM856914 | TYLCV-Gwangyang6 | GU325632.1 | TYLCV-Nons |
| HM856917 | TYLCV-Jangheung44 | JX961669.1 | TYLCV- Seongnam1-1 |
| KF225312 | TYLCV-Korea | JX961668.1 | TYLCV- Pyeongtaek1 |
| AB636411.1 | TYLCV-CJ-Ljs | JN183878.1 | TYLCV- Okcheon1 |
| AB636409.1 | TYLCV-GJ | HM856918 | TYLCV-Nonsan1 |
| HQ260984.1 | TYLCV-CW | HM856916 | TYLCV-Iksan9 |
| KY111368.1 | TYLCV | HM130913 | TYLCV-Jeonju |
| GU325634.1 | TYLCV-Bos | HM130914 | TYLCV-Jeju |
| GQ141873.1 | TYLCV-Bus | MN520589.1 | TYLCV-Yunnan |
| JN680149.1 | TYLCV- Goseong | N183877.1 | TYLCV-Gongju49 |
| JX961667.1 | TYLCV-Hwasun29 | AB613209.1 | TYLCV-NS |
| JX961666.1 | TYLCV-Ganghwa2 | AB613208.1 | TYLCV-IS |
| JX961665.1 | TYLCV-Cheongwon2 | HM856910.1 | TYLCV-Buyeo12 |
| JQ013090.1 | TYLCV-Gh |  |  |
| JQ013089.1 | TYLCV-To |  |  |
| JN183880.1 | TYLCV-Andong2 |  |  |
| JN183879.1 | TYLCV-Andong1 |  |  |
| JN183876.1 | TYLCV-Damyang38 |  |  |
| JN183875.1 | TYLCV-Damyang34 |  |  |
| JN183874.1 | TYLCV-Damyon17 |  |  |
| JN183873.1 | TYLCV-Damyon1 |  |  |
| JN183872.1 | TYLCV-Damyon43 |  |  |
| JN680150.1 | TYLCV-Haenam |  |  |
| HM856913.1 | TYLCV-Gwangju30 |  |  |
| HM856909.1 | TYLCV-Busan8 |  |  |
| HM856873.1 | TYLCV-Samcheonpo1 |  |  |

**Table S2.** Percentage of pairwise alignment of 40 newly isolates within TYLCV-KG1 and KG2 in Korea by using SDT.

| **TYLCV group** | **GenBank no.** | **Isolate** | **Percentage of pairwise (%)** | |
| --- | --- | --- | --- | --- |
|  |  |  | **KG1** | **KG2** |
| KG3 | ON982176 | Danong5 | 99.23 | 97.27 |
|  | ON982177 | Danong14 | 99.38 | 97.41 |
|  | ON982178 | Danong37 | 99.34 | 97.78 |
|  | ON982179 | Danong30 | 98.83 | 97.85 |
|  | ON982188 | Danong31 | 96.98 | 96.18 |
|  | ON982175 | Danong26 | 98.54 | 96.80 |
|  | ON982187 | Danong35 | 97.92 | 96.10 |
|  | ON982180 | Danong33 | 98.47 | 98.07 |
|  | ON982181 | Danong34 | 98.72 | 98.32 |
|  | ON982186 | Danong40 | 98.58 | 98.32 |
|  | ON982182 | Danong15 | 98.8 | 98.29 |
|  | ON982184 | Danong17 | 98.76 | 98.32 |
|  | ON982183 | Danong16 | 98.72 | 98.21 |
|  | ON982185 | Danong24 | 98.65 | 98.14 |
| KG4 | ON982189 | Danong29 | 93.56 | 92.36 |
|  | ON982190 | Danong39 | 93.70 | 92.43 |
|  | ON982193 | Danong3 | 94.00 | 92.94 |
|  | ON982192 | Danong6 | 93.05 | 91.41 |
|  | ON982191 | Danong23 | 94.00 | 92.80 |
|  | ON982194 | Danong21 | 94.25 | 93.09 |
|  | ON982195 | Danong12 | 94.40 | 93.12 |
|  | ON982196 | Danong10 | 94.40 | 92.98 |
|  | ON982200 | Danong32 | 94.47 | 92.90 |
|  | ON982199 | Danong19 | 94.47 | 92.90 |
|  | ON982197 | Danong7 | 94.32 | 92.76 |
|  | ON982198 | Danong8 | 94.36 | 92.83 |
| KG5 | ON982201 | Danong27 | 93.92 | 93.12 |
|  | ON982202 | Danong2 | 93.27 | 92.03 |
|  | ON982203 | Danong13 | 93.23 | 92.03 |
|  | ON982204 | Danong1 | 93.34 | 92.10 |
|  | ON982205 | Danong11 | 92.18 | 92.36 |
|  | ON982206 | Danong25 | 92.32 | 92.40 |
|  | ON982207 | Danong18 | 92.29 | 92.47 |
|  | ON982208 | Danong20 | 92.80 | 92.36 |
|  | ON982209 | Danong28 | 92.98 | 92.25 |
|  | ON982210 | Danong36 | 92.90 | 92.32 |
|  | ON982211 | Danong4 | 93.22 | 92.50 |
|  | ON982212 | Danong9 | 93.01 | 92.36 |
|  | ON982213 | Danong22 | 92.87 | 92.29 |
|  | ON982214 | Danong38 | 92.94 | 92.29 |

**Table S3**. TYLCV symptom severity score and viral copies number in this study at day post inoculation (dpi).

| Genotype | Symptoms score (mean of ten tomato plants ± SD) | | | |
| --- | --- | --- | --- | --- |
|  | 7 dpi | 14 dpi | 21 dpi | 28 dpi |
| TYLCV-KG1 | 0.7±0.2 | 1.2±0.2 | 2.3±0.2 | 3.2±0.2 |
| TYLCV-KG2 | 0.3±0.1 | 0.4±0.1 | 0.5±0.2 | 0.7±0.2 |
| TYLCV-KG3 | 0.8±0.3 | 1.6±0.2 | 2.8±0.3 | 3.8±0.2 |
| TYLCV-KG4 | 1.0±0.0 | 1.8±0.2 | 3.2±0.2 | 4.0±0.0 |
| TYLCV-KG5 | 0.3±0.1 | 0.2±0.0 | 0.5±0.0 | 0.7±0.2 |
| Genotype | Viral copies number (mean ± SD) | | | |
|  | 7 dpi | 14 dpi | 21 dpi | 28 dpi |
| TYLCV-KG1 | 6.44±0.48 x 10^5^ | 9.61±0.78 x 10^6^ | 3.85±1.93 x 10^7^ | 4.01±1.12 x 10^7^ |
| TYLCV-KG2 | 5.24±0.67 | 4.07±0.44 | 18.40±3.00 | 31.83±3.49 |
| TYLCV-KG3 | 6.74±0.18 x 10^6^ | 3.65±0.37 x 10^7^ | 7.07±0.64 x 10^7^ | 1.46±0.85 x 10^8^ |
| TYLCV-KG4 | 2.07±0.83 x 10^6^ | 2.24±0.37 x 10^7^ | 2.08±0.54 x 10^8^ | 1.82±0.64 x 10^8^ |
| TYLCV-KG5 | 10.17±1.60 | 20.07±4.98 | 62.52±14.31 | 310.34±52.32 |

**Table S4**. TYLCV viral copies number on breeding lines in this study at day post inoculation (dpi).

| Genotype | Breeding line | Symptoms score (mean of ten tomato plants ± SD) | |
| --- | --- | --- | --- |
|  |  | 7 dpi | 14 dpi |
| TYLCV-KG1 | Susceptible | 3.03 x 10^8^±3.10 x 10^7^ | 1.57 x 10^9^±1.19 x 10^8^ |
|  | *Ty*-1 | 512.01±103.16 | 2156.59±707.77 |
|  | *Ty*-2 | 1329.37±137.73 | 1293.38±201.48 |
| TYLCV-KG3 | Susceptible | 1.17 x 10^8^±3.21 x 10^7^ | 1.84 x 10^9^±4.86 x 10^8^ |
|  | *Ty*-1 | 4.60 x 10^6^±5.74 x 10^5^ | 1.41 x 10^8^±1.71 x 10^7^ |
|  | *Ty*-2 | 9232.16±1315.15 | 1.73 x 10^9^±3.44 x 10^8^ |
| TYLCV-KG4 | Susceptible | 3.30 x 10^6^±2.34 x 10^5^ | 1.99 x 10^9^±2.54 x 10^8^ |
|  | *Ty*-1 | 4.88 x 10^4^±6.31 x 10^3^ | 2.19 x 10^7^±5.09 x 10^6^ |
|  | *Ty*-2 | 1.01 x 10^5^±1.45 x 10^4^ | 1.41 x 10^4^±8.33 x 10^3^ |

**Table S5.** The details of TYLCV 40 newly isolates in this study**.**

| **TYLCV Group** | **Isolate (Danong)** | **Region** | **Cultivar** |
| --- | --- | --- | --- |
| KG3 | 5 | Daegok-ri, Dogok-myeon, Hwasun-gun, Jeollanam-do | Green tomatoes |
|  | 14 | Namjeong-ri, Neung-myeon, Hwasun-gun, Jeollanam-do | Jujube (holiday) |
|  | 15 | Subuk-myeon, Damyang-gun, Jeollanam-do | Jujube (damoa) |
|  | 16 | Subuk-myeon, Damyang-gun, Jeollanam-do | Full ripeness (pink type) |
|  | 17 | Bongsan-myeon, Damyang-gun, Jeollanam-do | Jujube |
|  | 24 | Gwangsan-gu, Gwangju | Jujube |
|  | 26 | Sinmun-dong, Gimhae-si, Gyeongnam | Full ripeness (pink type_Amos TY) |
|  | 30 | Haman-gun, Gyeongnam | Full ripeness (pink type _ Charmant) |
|  | 31 | Gwangyang, Jeollanam-do | Full ripeness (red _ red kan) |
|  | 33 | Sacheon, Jeollanam-do | Full ripeness (red system _ Scaletto) |
|  | 34 | Jeonnam Jinju | Jujube (TY Candy) |
|  | 35 | Seongseong-myeon, Boseong-gun, Jeollanam-do | Jujube |
|  | 37 | Sedo-myeon, Buyeo-gun, Chungcheongnam-do | Jujube (The Hard) |
|  | 40 | Sinbuk-eup, Chuncheon-si, Gangwon-do | Jujube (black jujube) |
| KG4 | 3 | Daeseo-myeon, Goheung-gun, Jeollanam-do | Full ripeness (Red type_Red Khan) |
|  | 6 | Daegok-ri, Dogok-myeon, Hwasun-gun, Jeollanam-do | Jujube |
|  | 7 | Daegok-ri, Dogok-myeon, Hwasun-gun, Jeollanam-do | Full ripeness (pink type) |
|  | 8 | Daegok-ri, Dogok-myeon, Hwasun-gun, Jeollanam-do | Jujube (TY Nonari) |
|  | 10 | Dogok-myeon, Hwasun-gun, Jeollanam-do | Full ripeness (red type) |
|  | 12 | Eori, Cheongpungmyeong, Hwasun-gun, Jeollanam-do | Jujube (Snacktini) |
|  | 19 | Noan-myeon, Naju-si, Jeollanam-do | Full ripeness (pink type) |
|  | 21 | Seoho-myeon, Yeongam-gun, Jeollanam-do | Full ripeness (pink type) |
|  | 23 | Nam-myeon, Jangseong-gun, Jeollanam-do | Jujube |
|  | 29 | Daesan-myeon, Changwon-si, Gyeongnam | Full ripeness (red line_TSmax) |
|  | 32 | Gwangyang, Jeollanam-do | Full ripeness (pink _ Mamilio) |
|  | 39 | Sedo-myeon, Buyeo-gun, Chungcheongnam-do | Jujube (Hot Tip) |
| KG5 | 1 | Pyeong-ri, Hwacheon-myeon, Hwasun-gun, Jeollanam-do | Jujube (The Hard) |
|  | 2 | Pumpyeong-ri, Iyang-myeon, Hwasun-gun, Jeollanam-do | Jujube (TY Nonari) |
|  | 4 | Pyeong-ri, Dogok-myeon, Hwasun-gun, Jeollanam-do | Jujube (Sun Power) |
|  | 9 | Dogok-myeon, Hwasun-gun, Jeollanam-do | Jujube |
|  | 11 | Daesan-myeon, Gochang-guk, Jeollabuk-do | Jujube |
|  | 13 | Hyosan-ri, Dogok-myeon, Hwasun-gun, Jeollanam-do | Jujube (Bettatini) |
|  | 18 | Songchon-dong, Naju-si, Jeollanam-do | Jujube |
|  | 20 | Gunseo-myeon, Yeongam-gun, Jeollanam-do | Jujube |
|  | 22 | Jangseong-eup, Jangseong-gun, Jeollanam-do | Jujube |
|  | 25 | Sinmun-dong, Changwon-si, Gyeongnam | Jujube (The Hard) |
|  | 27 | Sinmun-dong, Gimhae-si, Gyeongnam | Jujube (Best Honey) |
|  | 28 | Changnyeon-gun, Gyeongnam | Full ripeness (Red type_Red Khan) |
|  | 36 | Bongsan-myeon, Damyang-gun, Jeollanam-do | Jujube (TY Tiny) |
|  | 38 | Yeonmu-eup, Nonsan-si, Chungcheongnam-do | Jujube (TY Nonari) |

**Table S6.** Primer sets used for amplification and detection of viral DNA infecting-tomato in this study.

| Primer name | Nucleotide sequence (5’-3’) | Tm (℃) | Target size (bp) |
| --- | --- | --- | --- |
| Detection PCR |  |  |  |
| TYLCV-det-F | AAGCGACCAGGCGATATAATC | 57 | 1158 |
| TYLCV-det-R | AGGGGAACTCATCACTGCTC | 58 |  |
| TYLCKaV-det1K-F | GTGGGATCCATTGCTACACC | 60.5 | 1000 |
| TYLCKaV-det1K-R | GGCGTTGGGGATTCACAAG | 59.5 |  |
| ToLCNDV-det1K-F | GTGCAGTTGTCCCCATTGC | 59.5 | 1000 |
| ToLCNDV-det1K-R | CTGCGGAAAGCCCTGGG | 59.5 |  |
| TYLCTHV-det1K-F | CCGTCTGAACTTCGACAGC | 59.5 | 1000 |
| TYLCTHV-det1K-R | GAGGAACCACGACATAATCAC | 59.5 |  |
| Full length sequencing |  |  |  |
| 2.7K-TYLCV-F | GATATTAAGCATACTGGTATTGTTCGTTG | 57 | 2774 |
| 2.7K-TYLCV-R | TCATCCCGTTGCTCATAAGAC | 57 |  |
| Real-time PCR |  |  |  |
| qPCR-TYLCV-V1-F | CTCTGGAATGAAGGAACAGGC | 60 | 141 |
| qPCR-TYLCV-V1-R | GAGGCATGCGTACATGCCATATAC | 60 |  |
| EF1α-F | ATTGGAAACGGATATGCCCCT | 62.5 | 188 |
| EF1α-R | TCCTTACCTGAACGCCTGTCA | 62.8 |  |

**Table S7.** The list of additional begomovirus isolates and sequences used for sequence analysis and phylogenetic tree**.**

| **GenBank no.** | **Isolate** |
| --- | --- |
| KT992052.1 | Sweet potato leaf curl virus isolate 55-SPLCV-II |
| KU569600.1 | Tomato yellow leaf curl Kanchanaburi virus isolate TH14E1-3 segment DNA-A |
| KU569603.1 | Tomato yellow leaf curl Kanchanaburi virus isolate TH14E3-9 segment DNA-A |
| MN437658.1 | Squash leaf curl China virus isolate KN49A segment DNA-A |
| MN437662.1 | Squash leaf curl China virus isolate CRI136 segment DNA-A |
| KT426907.1 | Tomato leaf curl New Delhi virus isolate RG5 |
| LN886524.1 | Tomato leaf curl New Delhi Virus complete genome clone 4NA3-6RS28 |
| MZ054162.1 | Pepper yellow leaf curl Thailand virus isolate KB segment A |
| MK946436.1 | Pepper yellow leaf curl Thailand virus isolate BRM103 segment DNA-A |
| JX679252.1 | Tomato yellow leaf curl China virus isolate SC226-8 |
| KU934106.1 | Tomato yellow leaf curl China virus isolate YN4475 |
| KT992047.1 | Tobacco leaf curl virus isolate KH7 |
| HM164549.1 | Tobacco leaf curl virus isolate TLCV-Korea-SC2 |

**Table S8**. Primer sets used for amplification of TYLCV infectious clone newly isolates in this study.

| Target amplification | Primer name | Nucleotide sequence (5’-3’) | Target size (bp) |
| --- | --- | --- | --- |
| TYLCV-IC-KG3 | IC1KG3-F | GGTACCGTTGAAATGAATCGGTGTCCC | 1186 |
|  | IC1KG3-R | AGAGGCATGCGTACATGCCATATACAGTAAC |  |
|  | IC2KG3-F | GTACGCATGCCTCTAATCCAGTGTATGCAAC | 1920 |
|  | IC2KG3-R | GGATCCATTGCAAGACAAAAAACTTGGGG |  |
| TYLCV-IC-KG4 | IC1KG4-F | GGTACCATTTACAAATATGCCCTTGTACC | 1186 |
|  | IC1KG4-R | CGAGGCATGCGTACATGCCATATACAGTAAC |  |
|  | IC2KG4-F | GTACGCATGCCTCGAATCCAGTGTATGCAAC | 1906 |
|  | IC2KG4-R | GGATCCATTGCAAGACAAAAAACTTGGGG |  |
| TYLCV-IC-KG5 | IC1KG5-F | GGTACCATGGTCAATGAGTACCGATTGAC | 1204 |
|  | IC1KG5-R | CGAGGCATGCGTACATGCCATATACAATAAC |  |
|  | IC2KG5-F | GTACGCATGCCTCGAATCCAGTGTATGCAAC | 1938 |
|  | IC2KG5-R | GGATCCATTGCAAGACAAAAAACTTGGGG |  |

**Table S9**. Primer sets used for RT-qPCR relative expression analysis of ORFs-TYLCV, *Ty*-1, and *Ty*-2 gene in this study.

| Target amplification | Primer name | Nucleotide sequence (5’-3’) |
| --- | --- | --- |
| ORFs-V1 | ORFs-V1-F | ctctggaatgaaggaacaggc |
|  | ORFs-V1-R | gaggcatgcgtacatgccatatac |
| ORFs-V2 | ORFs-V2-F | gagtttcctgaatctgttcacgg |
|  | ORFs-V2-R | tcgcttcgacatagtcacgg |
| ORFs-C1 | ORFs-C1-F | AACACAAAGTACGGGAAGCC |
|  | ORFs-C1-R | GAACAGTGGCTCGTAGAGG |
| ORFs-C4 | ORFs-C4-F | ccatccgaacattcaggcag |
|  | ORFs-C4-R | gttggcagattgctgacctc |
| *Ty*-1 | *Ty*-1-F | CTGGGCGTGTTTTGGTCTAC |
|  | *Ty*-1-R | CTCAGTAGCAGCTGACCTCG |
| *Ty*-2 | *Ty*-2-F | CCCTGCCAGCACTAGGACAGCTTTG |
|  | *Ty*-2-R | AATTCGGGACAATTTTGAATTAGAAC |
